# An ALS‐associated mutation in the C‐terminal α‐helix of TDP‐43 uncouples condensate formation and amyloid assembly

**DOI:** 10.1002/pro.70565

**Published:** 2026-04-13

**Authors:** Emily J. Byrd, Joel A. Crossley, Chalmers C. C. Chau, Paolo Actis, Antonio N. Calabrese

**Affiliations:** ^1^ Astbury Centre for Structural Molecular Biology, School of Molecular and Cellular Biology, Faculty of Biological Sciences University of Leeds Leeds UK; ^2^ School of Electronic and Electrical Engineering University of Leeds Leeds UK; ^3^ Bragg Centre for Materials Research University of Leeds Leeds UK

**Keywords:** amyloid assembly, biomolecular condensates, intrinsically disordered proteins, Ion mobility–mass spectrometry, TDP‐43 C‐terminal domain

## Abstract

TAR DNA‐binding protein 43 (TDP‐43) plays a critical role in RNA metabolism and is incorporated into biomolecular condensates called stress granules. In amyotrophic lateral sclerosis (ALS) and several other neurodegenerative disorders, TDP‐43 undergoes aberrant phase transitions, forming insoluble amyloid aggregates, including fibrils composed of solely its intrinsically disordered C‐terminal domain (CTD). Despite its central role in disease, the conformational dynamics of the CTD remain poorly understood due to its heterogeneous and transient conformational landscape. Here, we employ native ion mobility‐mass spectrometry (IM‐MS) using nanopipette sub‐micron nano electrospray ionization (nanoESI) emitters to characterize the conformational landscape of wild‐type and ALS‐associated TDP‐43 CTD variants (Q331K and R361S) under different solution conditions. Our data suggest that mutations and salt concentration modulate the CTD's conformations. Combined with thioflavin T fluorescence, light scattering, and microscopy, we reveal that these conformational shifts correlate with altered amyloid assembly kinetics and propensity to form condensates. Notably, the Q331K variant, which has a mutation in the transient α‐helical region in the CTD, has reduced propensity to form biomolecular condensates but can undergo amyloid assembly in the absence of condensate formation, suggesting that sequence alterations in this α‐helical region can tune the molecular mechanism of amyloid assembly. This study demonstrates the power of IM‐MS in probing disordered proteins and reveals mechanistic insights into how disease‐associated mutations differentially tune TDP‐43 CTD amyloid assembly mechanisms.

## 1 INTRODUCTION

**FIGURE 1:**
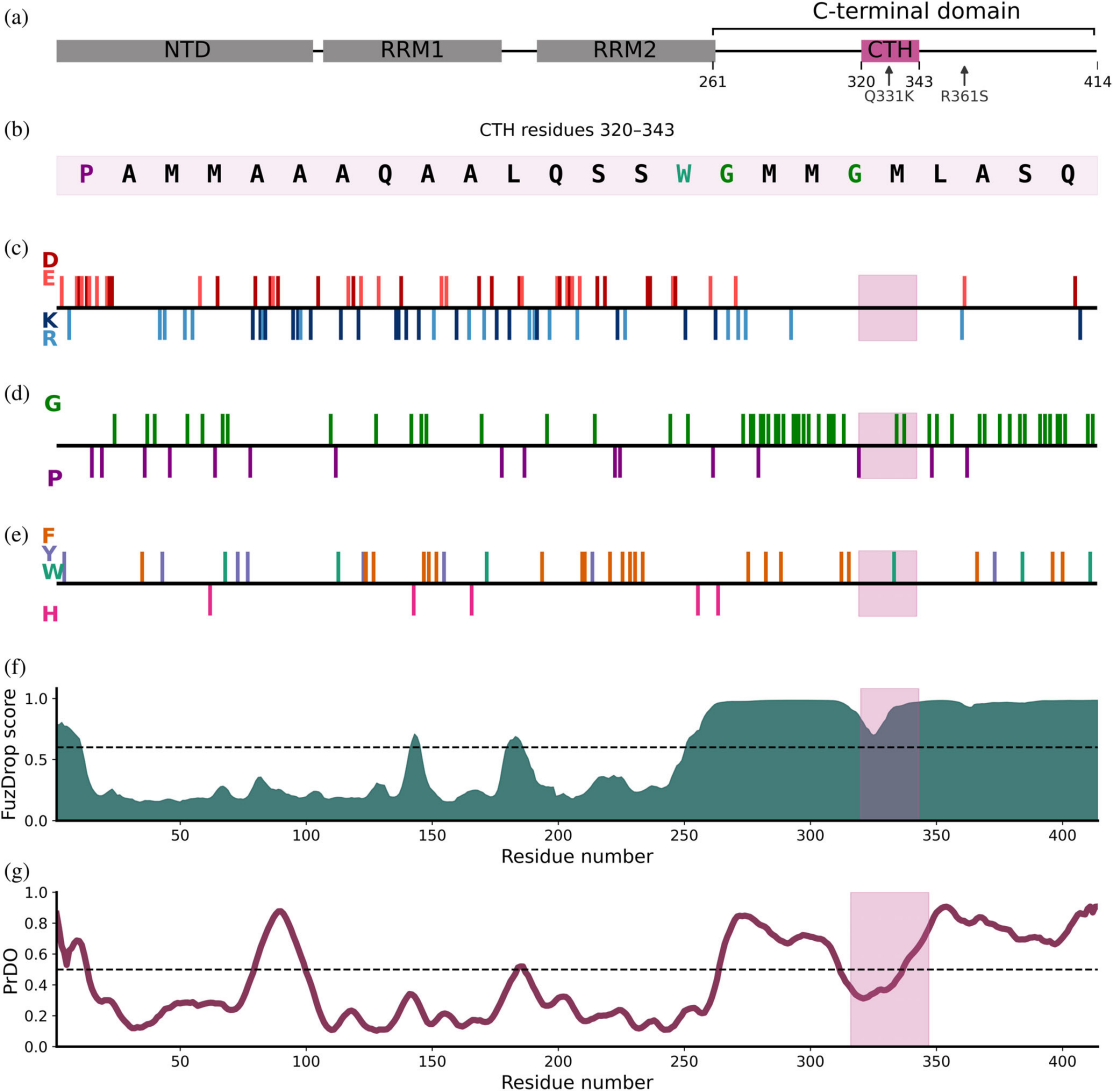
Bioinformatic analysis of the TAR DNA‐binding protein 43 (TDP‐43) protein sequence. (a) Schematic of the domain architecture of TDP‐43, showing the N‐terminal domain (NTD), RNA recognition motifs (RRM1 and RRM2) and the intrinsically disordered C‐terminal domain (CTD). The CTH is highlighted in pink and the location of the Q331K and R361S mutations in the CTD are shown by arrows. (b) The sequence of the CTH. Residues are colored according to the scheme used in (c, d, and e). (c) Distribution of charged residues along the TDP‐43 sequence. Negatively charged residues (Asp, D/Glu, E) are shown in red and positively charged residues (Lys, K/Arg, R) are shown in blue; there are no arginine residues present in the CTD. (d) Distribution of Gly (G) and Pro (P) residues along the sequence, indicating flexible and structure‐breaking regions, respectively. (e) Distribution of Phe (F), Tyr (Y), Trp (W) and His (H) residues along the sequence. (f) Sequence‐based prediction to highlight regions of TDP‐43 that promote condensate formation, calculated using FuzDrop (Hardenberg et al., 2020; Hatos et al., 2022; Vendruscolo & Fuxreiter, 2022), with a score above 0.6 indicating a condensate driving region. (g) Disorder propensity plot generated by protein disorder prediction system (PrDOS) (Ishida & Kinoshita, 2007). Values above the 0.5 threshold (dashed line) indicate disordered regions, showing that the CTD residues 269–414 of TDP‐43 are predominantly intrinsically disordered. The transient α‐helical region (CTH) between residues ~320 and 343 is highlighted in pink, and comprises residues with scores less than 0.5, consistent with its ability to form an ordered structure (f).

TAR DNA‐binding protein 43 (TDP‐43) is an RNA‐binding protein that plays a central role in the regulation of RNA splicing, stability, transport, and translation (Khalfallah et al., 2018; Suk & Rousseaux, 2020). It is predominantly localized in the nucleus under basal conditions, but in response to cellular stress TDP‐43 migrates to the cytoplasm where it contributes to the formation of biomolecular condensates known as stress granules (Gasset‐Rosa et al., 2019; Lee et al., 2021). Condensate formation is a dynamic and reversible process that facilitates sub‐organellar compartmentalization (Alberti et al., 2019; Banani et al., 2017; Guseva et al., 2023; Shin & Brangwynne, 2017). While TDP‐43 condensate formation is believed to be part of a normal cellular stress response, aberrations in this process are implicated in neurodegenerative diseases, suggesting a switch from function to dysfunction (de Oliveira et al., 2019; Guseva et al., 2023; Wang et al., 2018). TDP‐43 is composed of 414 amino acids, comprising a structured N‐terminal domain followed by two RNA recognition motifs (RRMs), RRM1 and RRM2, which are connected by unstructured linkers (Figure 1a) (Cohen et al., 2011; Kuo et al., 2014; Prasad et al., 2019). The C‐terminal domain (CTD) is primarily intrinsically disordered and referred to as a low complexity domain (LCD) as it comprises primarily glycine and aromatic residues, as well as a distribution of proline residues known to promote condensate formation (Zhang et al., 2020), and it also lacks charged residues (Figure 1b–e) (Shenoy et al., 2023). The CTD is also thought to be a key driver of TDP‐43 condensate formation (Figure 1f), and it features a small, transient α‐helix (CTH; Figure 1b,g) which has been shown to undergo allosteric changes in dynamics upon RNA binding to RRM1 and RRM2 (Conicella et al., 2020; Minshull et al., 2024).

In pathological contexts, TDP‐43 undergoes irreversible liquid‐to‐solid transitions, forming insoluble cytoplasmic amyloid fibrils that are a defining hallmark of amyotrophic lateral sclerosis (ALS) and other TDP‐43 proteinopathies (Chien et al., 2021). These aggregates consist of full‐length TDP‐43 as well as truncated fragments comprising the C‐terminal region (the proteins are often hyperphosphorylated and ubiquitinated; Igaz et al., 2009). A range of C‐terminal fragments (CTFs) have been identified, including CTF‐16 which comprises the CTD in isolation (studied here), CTF‐25, a 25 kDa fragment comprising the CTD and part of RRM2, and a less frequently observed CTF‐35 which comprises the CTD, RRM2, RRM1 and part of the N‐terminal domain (NTD) (Berning & Walker, 2019; Shenoy et al., 2020). Fibril structures of both full‐length TDP‐43 and truncations comprising the entire CTD in vitro (residues approximately 267–414) (Prasad et al., 2019) have been determined using cryogenic electron microscopy (cryo‐EM) (Arseni et al., 2023, 2024; Cao et al., 2019; Li et al., 2021; Sharma et al., 2024). Structures of TDP‐43 amyloid fibrils isolated from the frontal and motor cortices of ALS and frontotemporal lobar degeneration (FTLD) patients have revealed distinct fibril polymorphs (Arseni et al., 2022, 2023), suggesting that specific amyloid folds are characteristic of different TDP‐43 proteinopathies. Recently, the structure of filaments from individuals with FTLD (type C) were determined and shown to be comprised of heteromeric amyloid filaments composed of TDP‐43 as well as a second protein ANXA11, a calcium‐dependent phospholipid binding protein (Arseni et al., 2024; Towle & Treadwell, 1992). Amyloid fibrils formed in vitro from the entire CTD of TDP‐43 revealed fibrils formed from a single protofilament comprised of a large 139‐residue amyloid core (Li et al., 2021). Additionally, TDP‐43 is known to undergo many different post‐translational modifications (PTMs) such as acetylation, SUMOylation, ubiquitination, methylation, citrullination, and phosphorylation which likely influence the function and dysfunction of the protein (Aikio et al., 2025; Cohen et al., 2015; Cracco et al., 2022; Inukai et al., 2008; Kellett et al., 2025; Neumann et al., 2006, 2009; Saunders et al., 2025).

Notably, the intrinsically disordered CTD of TDP‐43 is critical for both condensate formation and pathological amyloid assembly (Chang et al., 2020; Conicella et al., 2016, 2020; Lin et al., 2024). The CTD is enriched in glycine and aromatic residues (Figure 1c), harbors most of the known disease‐associated mutations, and is prone to amyloid formation (Arnold et al., 2013; Conicella et al., 2016; Neumann et al., 2009; Pesiridis et al., 2009; Suk & Rousseaux, 2020; Van Langenhove et al., 2012). The conformational flexibility of the CTD presents a major challenge for structural characterization, especially under physiological conditions. Nuclear magnetic resonance (NMR) spectroscopy has been applied to characterize the transient CTH in the monomeric TDP‐43 CTD and shown that the CTH (residues 320–343) becomes stabilized in dimeric assemblies (Rizuan et al., 2025). Additionally, G335 and G338 were identified as “helix terminators” and when mutated to alanine this results in enhanced condensate assembly, suggesting that helix enhancing mutations tune the TDP‐43 CTD's ability to form biomolecular condensates (Conicella et al., 2020). Amyloid assembly of the TDP‐43 CTD is known to proceed through condensate‐mediated or hydrogel‐mediated assembly (Babinchak et al., 2019). The addition of salt has been shown to promote TDP‐43 CTD condensate assembly by enhancing intermolecular contacts through electrostatic screening (including the protein backbone and termini as there are only three negatively charged residues in the CTD; Figure 1b), biasing the TDP‐43 CTD toward condensate‐driven amyloid assembly (Babinchak et al., 2019; Lin et al., 2024).

By contrast with the averaging which occurs in NMR spectroscopy experiments, native ion mobility‐mass spectrometry (IM‐MS) offers the ability to resolve distinct conformational states based on their rotationally averaged collision cross section (CCS), enabling the interrogation of heterogeneity within a conformational ensemble in vacuo with high sensitivity (Allison & Bechara, 2019; Beveridge & Calabrese, 2021; Marklund et al., 2015; Stuchfield & Barran, 2018). Proteins are ionized using nano electrospray ionization (nanoESI) under non‐denaturing conditions and kinetically trapped in their solution phase states, typically using a solution comprising volatile salts such as ammonium acetate to facilitate transfer into the gas phase (Fenn, 2003; Grandori et al., 2009). Under standard nanoESI conditions, salts such as Na^+^, K^+^, PO_4_^3−^ can cause ion suppression and adduct to protein ions, which reduces spectral resolution and sensitivity (Cassou & Williams, 2014; Metwally et al., 2015; Sterling et al., 2010; Susa et al., 2017). We, and others (Byrd, Norgate, et al., 2025; Drachman et al., 2024; Panczyk et al., 2020; Susa et al., 2017, 2018; Yuill et al., 2013), have recently demonstrated the use of submicron nanoESI “nanopipette” emitters (Drachman et al., 2024; Panczyk et al., 2020; Susa et al., 2017, 2018; Yuill et al., 2013) which enable nanoESI analysis in biochemical buffers and under elevated salt concentrations.

Here, we use submicron nanoESI emitters to investigate the effect of increasing ionic strength on the CCS distributions of the TDP‐43 CTD and two ALS‐associated TDP‐43 CTD variants (Q331K and R361S) as measured by traveling wave IM‐MS in nitrogen gas (^TW^CCS_N2_), to elucidate how solution conditions tune the structural properties of the monomeric TDP‐43 CTD. The Q331K mutation lies within a region that adopts α‐helical structure (Figure 1a), whereas the R361S mutation is present outside of the α‐helix (Li et al., 2018; Watkins et al., 2021). These two variants enabled us to understand how modulating the structured CTH element within the otherwise low sequence complexity CTD tunes the structural and functional properties of TDP‐43 CTD. In combination with nanopipette nanoESI emitters, we interrogated the role of NaCl in tuning TDP‐43 CTD conformations. To contextualize the conformational changes observed via IM‐MS, we complemented our mass spectrometry data with multiple orthogonal biophysical assays. We employed thioflavin T (ThT) fluorescence assays to monitor amyloid assembly kinetics, while light scattering (nephelometry) and differential interference contrast (DIC) microscopy provided insights into condensate formation. Our results reveal that elevated salt concentrations induce measurable shifts in the CTD conformational ensemble, as reflected by changes in its CCS distributions. These conformational shifts correlate with increased amyloidogenicity for Q331K TDP‐43 CTD. The Q331K mutation, present within the C‐terminal α‐helix, reduces the propensity for condensate formation under the conditions tested, but this variant can still form amyloid fibrils under elevated salt concentrations in the absence of condensates. These findings highlight the role of electrostatic interactions in modulating the balance between condensate formation and pathological amyloid assembly and demonstrate how a single amino acid substitution can tune the self‐assembly mechanism of TDP‐43. Taken together, our results highlight the critical role of the CTH region in governing the route of amyloid assembly for the TDP‐43 CTD and demonstrate how a single mutation in this region can alter the mechanism of amyloid assembly.

## 2 RESULTS AND DISCUSSION

### 2.1 Salt‐driven expansion of the wild‐type CTD facilitates condensate assembly

**FIGURE 2:**
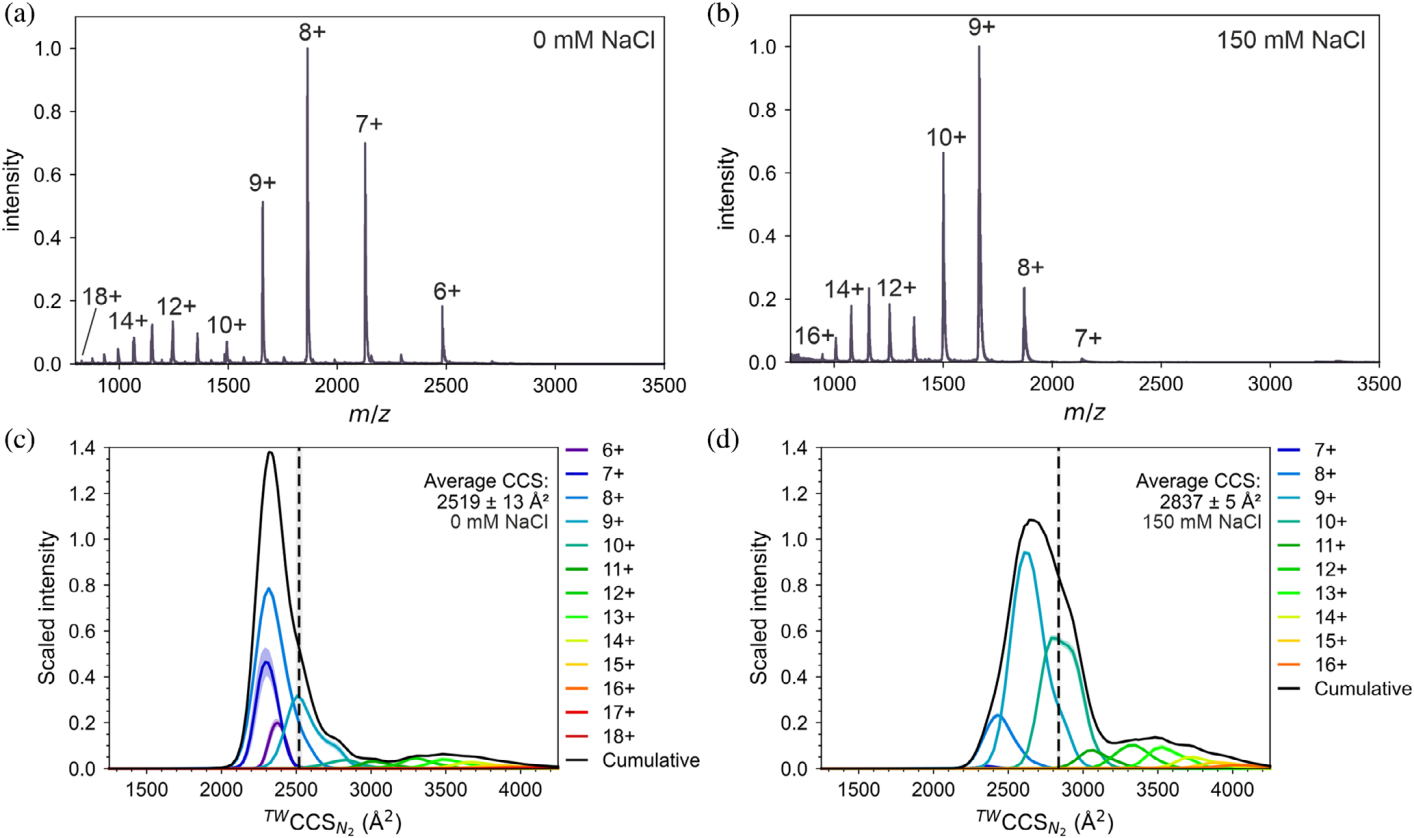
Salt‐induced expansion of the TAR DNA‐binding protein 43 (TDP‐43) C‐terminal domain (CTD) revealed by native mass spectrometry (MS), ion‐mobility MS. (a) Native nanoESI mass spectrum of wild‐type (WT) TDP‐43 CTD acquired in 20 mM ammonium acetate (pH 5.5) shows a broad charge‐state distribution (6+ to 18+). (b) Native nanoESI mass spectrum of WT TDP‐43 CTD acquired in 20 mM ammonium acetate (pH 5.5) with 150 mM NaCl, acquired using submicron nanopipette nanoESI emitters (Byrd, Norgate, et al., 2025). (c) Cumulative ^TW^CCS_N2_ distribution, showing the summed data for each charge state in 20 mM ammonium acetate (pH 5.5), the shaded region represents the range of three replicates. The cumulative trace (black) yields an average ^TW^CCS_N2_ of 2519 Å^2^ (dashed line) with an error of ± 13 Å^2^ giving a range of 2506–2532. (d) In 20 mM ammonium acetate (pH 5.5) with 150 mM NaCl the ^TW^CCS_N2_ distribution shift to larger values with a broader distribution, giving an average of 2837 Å^2^ with an error of ±5 Å^2^ giving a range of 2832–2842. The gray shaded region is the standard error from *n* = 3.

Native mass spectrometric analysis of the wild‐type (WT) TDP‐43 CTD suggests that the protein populates two major conformational families, as revealed by its bimodal charge state distribution (Figure 2a). As the charge state distribution of a protein in a native IM‐MS experiment reflects its solvent accessible surface area (Grandori et al., 2009), we describe these two conformational families as representing more expanded conformers (charge states 18+ to 10+) and more compact states (charge states 9+ to 6+). The bimodal distribution of charge states and higher relative intensity of the more compact conformational family (~70% of the measured signal) was surprising, suggesting that the TDP‐43 CTD preferentially undergoes compaction, likely due to intramolecular interactions, under the conditions employed (Figure 2a). This is strikingly different from the native mass spectra of other intrinsically disordered proteins (IDPs), such as N‐terminally‐acetylated α‐synuclein, which has a similar mass to TDP‐43 CTD (α‐synuclein: 14,502 Da, TDP‐43 CTD: 14,889 Da) but favors more extended conformers (Byrd et al., 2023). A similar phenomenon of overly compact disordered conformations has been described previously using NMR and Förster resonance energy transfer (FRET) for eukaryotic translation initiation factor elF4B, an IDP which participates in multivalent interactions (Swain et al., 2024).

We, and others, have shown previously that ionic strength can modulate the structure and dynamics of IDPs and that these effects can be deduced by native MS (Byrd, Norgate, et al., 2025; Susa et al., 2017; Xia et al., 2019). While the mass spectra presented here were acquired under low ionic strength conditions (20 mM ammonium acetate, pH 5.5), raising the ionic strength by addition of 150 mM NaCl (Conicella et al., 2016; Rizuan et al., 2025) shifts the charge state distribution of the TDP‐43 CTD toward higher charges (dominant 8+ to 10+; Figure 2b), indicating salt‐driven chain expansion. A similar electrostatic‐induced expansion has been observed previously for the protein ubiquilin‐2 (UBQLN2), which forms condensates under physiological conditions (Robb et al., 2023). Additionally, recent findings using native MS highlighted that the relative disorder content of a protein can be determined from its charge state distribution and, in the absence of ionizable residues such as lysine and arginine, the charge state distribution is dictated by the solvent accessible surface area, with the backbone amines and protein termini acting as charge carriers (Osterholz et al., 2025). This is particularly relevant for LCDs such as the TDP‐43 CTD which contains few K/R residues (Figure 1c). Twenty millimolar ammonium acetate (pH 5.5) was selected as a standard native MS‐compatible solution to preserve non‐covalent interactions while minimizing electrostatic screening. We have previously used 20 mM ammonium acetate for studies on α‐synuclein, which is an IDP of similar molecular weight (14.5 kDa) to the TDP‐43 CTD (14.6 kDa) (Byrd et al., 2023; Byrd, Rowlinson, et al., 2025). Although not physiologically representative, these conditions enable interrogation of the intrinsic conformational ensemble of the CTD prior to ionic‐strength modulation, and act as a control condition. Addition of 150 mM NaCl was used to approximate physiological ionic strength, enabling assessment of electrostatic screening effects under near‐cellular ionic strength conditions (Stein et al., 1979). For these experiments we also used mildly acidic conditions, which have been shown to reduce spontaneous TDP‐43 aggregation and improve monomer stability prior to assembly assays (Dang et al., 2021).

To further investigate the structural properties of TDP‐43 CTD, we used IM‐MS to measure the CCS of each ion species. Data from traveling wave ion mobility in nitrogen gas (^TW^CCS_N2_) demonstrate that the global CCS distribution (CCSD) of TDP‐43 is surprisingly monodisperse, suggesting that the TDP‐43 CTD conformational landscape is tightly restricted (average ^TW^CCS_N2_ of 2519 Å^2^; Figure 2c). However, it should be noted that additional, more extended conformations are observed at low abundance in the CCSD (between 3000 and 4000 Å^2^, Figure 2c). Addition of 150 mM NaCl resulted in an increase in the average ^TW^CCS_N2_ of the WT TDP‐43 CTD (~13% increase to 2837 Å^2^), consistent with the added NaCl mediating expansion of the TDP‐43 CTD chain. The CCSDs in the presence of added NaCl are also wider (Figure 2c,d), suggesting that a more diverse array of conformers is co‐populated.

It is important to note that the conformation of a protein can impact its ionization pathway during nanoESI, which may influence the observed gas‐phase conformations. Native/globular proteins with lower charge states have been proposed to ionize in a process described by the charged residue model (CRM), in which analytes are desolvated from shrinking droplets and closely retain solution‐like globular conformations in vacuo. In contrast, highly charged species are thought to arise, at least in part, from chain ejection mechanisms (CEM), whereby extended regions are expelled from droplets, potentially yielding elongated gas‐phase conformations with increased surface area that may exceed their solution dimensions (Konermann et al., 2013; Pimlott & Konermann, 2021). Accordingly, while we include the full charge state distribution to illustrate population redistribution across conditions, it is important to recognize that the absolute CCS values of the most highly charged states should be interpreted with some caution. Importantly, the salt‐dependent conformational expansion we observe is clearly evident within the dominant low‐charge (compact) ensemble (6+ to 10+), supporting the notion that the reported ensemble shift upon NaCl addition reflects a genuine change in solution behavior rather than being solely a result of the nanoESI process.

Our data are consistent with a model whereby salt induced electrostatic screening disrupts long‐range intramolecular interactions that mediate TDP‐43 CTD structural collapse to tune the conformational landscape of monomeric TDP‐43 CTD. This conformational remodeling could aid in the TDP‐43 CTD forming intermolecular contacts that are critical for stabilizing condensates (Poudyal et al., 2023) as elevated NaCl has been shown previously by others to increase the propensity of several proteins to form condensates (Duan & Wang, 2024; Sternke‐Hoffmann et al., 2024; Sun et al., 2019).

### 2.2 The Q331K mutation uncouples condensate formation from amyloid assembly

Next, we set out to explore if the CTH sequence (residues 320–343) plays a role in regulating the conformational ensemble of TDP‐43 CTD. We therefore designed a comparative study employing a TDP‐43 CTD variant containing a known sporadic ALS‐associated mutation (Q331K) (Arnold et al., 2013; Conicella et al., 2016, 2020). As a control, we also explored the effect of another known sporadic ALS‐associated mutation distal to the CTH, R361S (Johnson et al., 2009).

**FIGURE 3:**
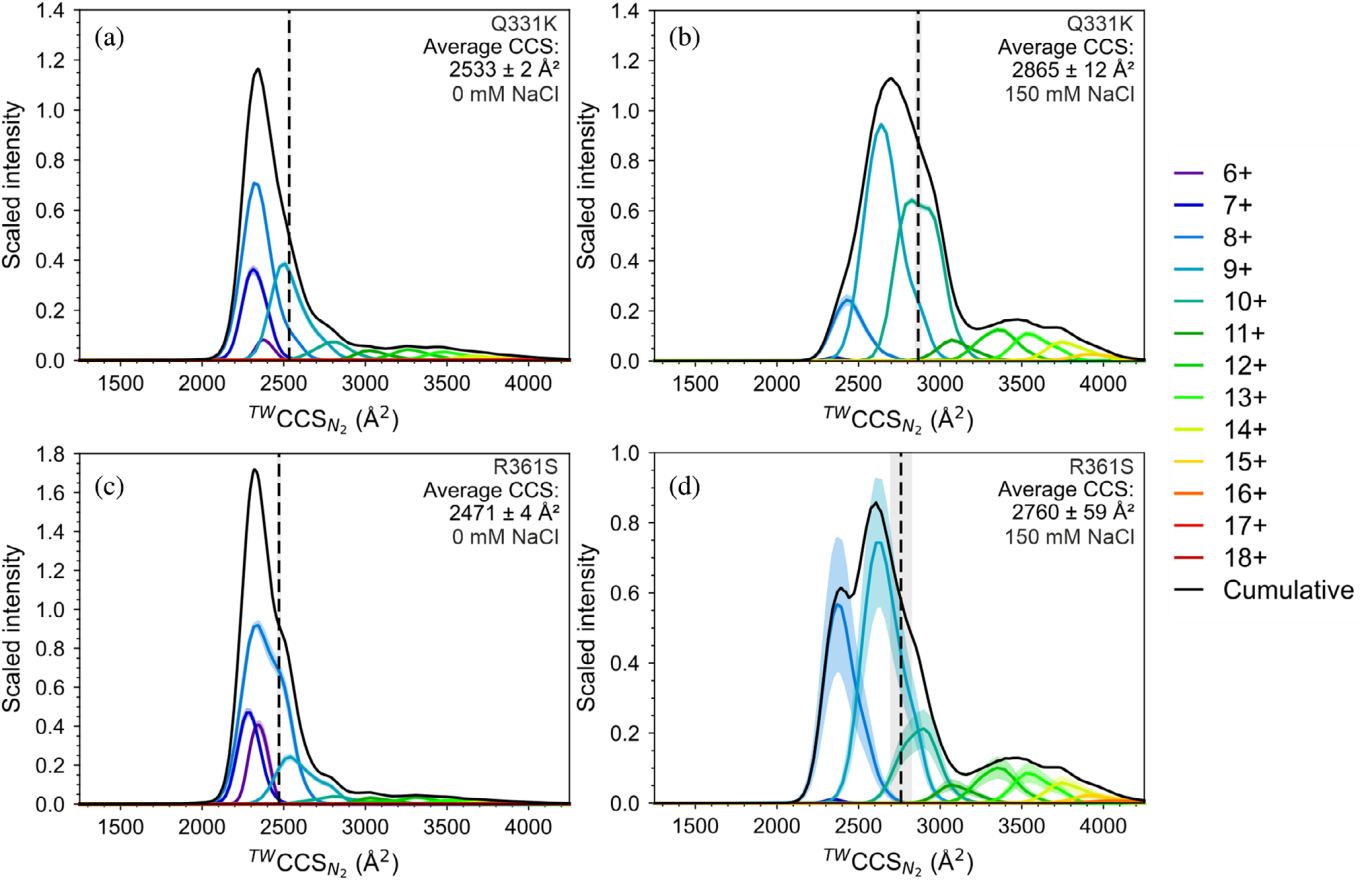
Salt induced expansion of amyotrophic lateral sclerosis (ALS)‐associated variants of the TAR DNA‐binding protein 43 (TDP‐43) C‐terminal domain (CTD) revealed using IM‐MS. ^TW^CCS_N2_ ion mobility distributions for the Q331K TDP‐43 CTD variant in (a) 20 mM ammonium acetate (pH 5.5) or (b) 20 mM ammonium acetate (pH 5.5) with 150 mM NaCl. ^TW^CCS_N2_ ion mobility distributions for the R361S TDP‐43 CTD variant in (c) 20 mM ammonium acetate (pH 5.5) or (d) 20 mM ammonium acetate (pH 5.5) with 150 mM NaCl. In (a–d), the cumulative trace (black) yields an average ^TW^CCS_N2_ of the distribution plotted as a dashed black line. The gray shaded region is the standard error from *n* = 3.

First, we applied IM‐MS to test whether the two TDP‐43 CTD variants have globally similar CCSDs to the WT protein. The CCSDs of all three proteins were broadly similar, with average CCS values of WT: 2519 Å^2^, Q331K: 2533 Å^2^ and R361S: 2471 Å^2^ in the absence of added NaCl (Figures 2c and 3a,c). Like WT TDP‐43 CTD, the native mass spectra and IM‐MS derived CCSDs of both variants in the presence of 150 mM NaCl suggest a conformational expansion is occurring upon addition of NaCl (~13% increase for all proteins; Figures 2c,d and 3a–d). A shift toward higher charge states is also observed in the native mass spectra upon addition of 150 mM NaCl (Figure S1). Combined, these data suggest that the overall conformational ensemble of WT TDP‐43 CTD and the two variants studied are comparable both at low and high ionic strength.

**FIGURE 4:**
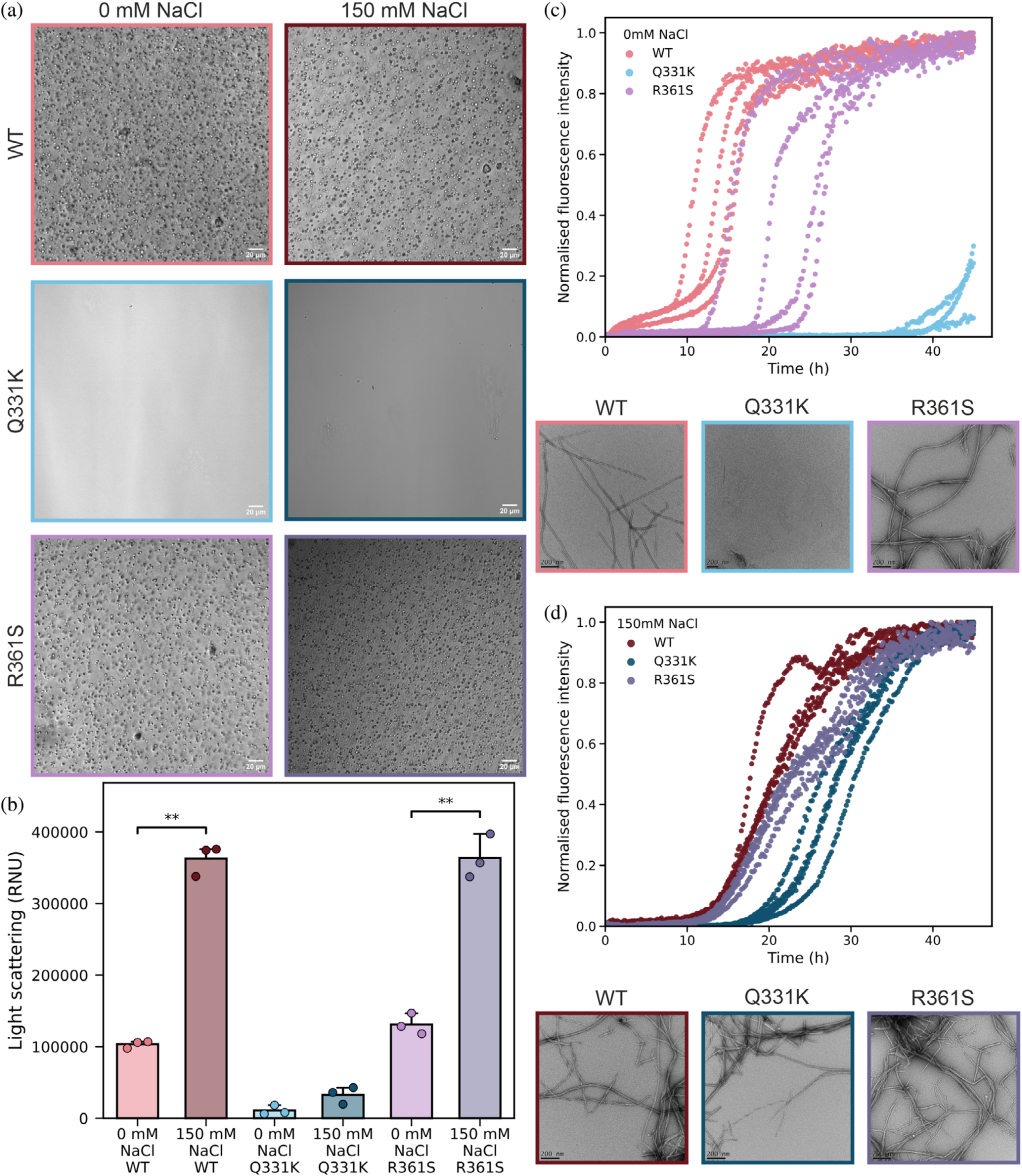
Point mutations and ionic strength differentially tune TAR DNA‐binding protein 43 (TDP‐43) C‐terminal domain (CTD) condensate formation and amyloid assembly. (a) Differential‐interference‐contrast images of the wild‐type (WT) TDP‐43 CTD in 0 mM NaCl (light red) and 150 mM NaCl (dark red), the Q331K TDP‐43 CTD in 0 mM NaCl (light blue) and 150 mM NaCl (dark blue), the R361S TDP‐43 CTD in 0 mM NaCl (light purple) and 150 mM NaCl (dark purple). Scale bars = 20 μm. Protein concentration was 50 μM measured in 20 mM ammonium acetate, pH 5.5. (b) Nephelometry light scattering measurements (*n* = 3) after 10‐h incubation. An unpaired two‐sided Welch's *T*‐test was performed for significance where ** = *p* < 0.01. (c, d) Thioflavin T (ThT) fluorescence kinetics of amyloid formation at 0 mM (c) and 150 mM (d) NaCl (*n* = 4) (upper panels). The insets (lower panels) are representative transmission electron microscopy (TEM) images which were taken at the timepoint where WT reached maximal fluorescence (scale bar = 200 nm) showing amyloid fibrils recovered at the end points of the assembly reactions.

Differential interference‐contrast (DIC) microscopy confirmed that WT and R361S TDP‐43 CTD readily formed micron‐sized condensates in both the absence of NaCl and the presence of 150 mM NaCl under the solution conditions tested (Figure 4a). Q331K TDP‐43 CTD did not form condensates under either solution condition tested (Figure 4a). Given this surprising observation, we next sought to further explore the condensation propensities of the Q331K variant to determine whether condensates could be observed with increasing concentrations of protein/NaCl (Figures S2–S5). For the WT TDP‐43 and R361 TDP‐43 CTD variant, condensate formation was observed under all conditions tested (Figures S2, S3, and S5). In the case of the Q331K variant, condensate formation was impaired, with condensates only observed at elevated protein/NaCl concentrations (in 50 mM NaCl, a 200 μM protein concentration was required, whereas in 150 mM NaCl condensate formation was observed at protein concentrations exceeding 100 μM) (Figures S2 and S4).

These qualitative findings were quantitatively confirmed using nephelometry (Figure 4b), where high levels of light scattering were observed for both WT and R361S TDP‐43 CTD under both low and elevated NaCl conditions. Conversely, low levels of light scattering were measured for Q331K TDP‐43 under both low and elevated NaCl conditions, consistent with the notion that this mutation abrogates condensate formation under these conditions. Intriguingly, the measured light scattering increased for both WT and R361S TDP‐43 CTD when the NaCl concentration was elevated (Figure 4b) suggesting either increased propensity to form condensates and/or altered condensate size/morphology in the presence of added NaCl.

Next, we sought to determine whether the TDP‐43 CTD variants retain the ability to assemble into amyloid fibrils by performing ThT fluorescence assays under identical solution conditions to those used above. ThT interacts with the cross‐β structure, characteristic of amyloid fibrils through the hydrogen bonding network of stacked β‐sheets which results in the immobilization of the rotational freedom of ThT molecules and subsequently fluorescence (Biancalana et al., 2009; Groenning et al., 2007) (note that ThT is also known to interact with oligomeric species containing β‐sheet content such as β‐barrel structures (Bieschke et al., 2005; Maezawa et al., 2008), but here we refer to ThT kinetics being reflective of amyloid fibril assembly). Interestingly, in the absence of NaCl the Q331K variant showed a pronounced reduction in the rate of amyloid assembly compared to WT and R361S TDP‐43 CTD (Figure 4c,d) with an increase in ThT fluorescence observed after around 40 h, consistent with delayed aggregation by this variant. Note that there is some degree of variation of kinetics observed for R361S TDP‐43 CTD (Figure 4c) which we have not explored further, and we postulate may be due to heterogeneity in the degree of condensate assembly in different sample wells. The addition of 150 mM NaCl rescued the ability of Q331K TDP‐43 CTD to form amyloid fibrils (Figure 4c,d), despite the protein not forming condensates under these conditions. Interestingly, the WT TDP‐43 CTD appeared to exhibit slower amyloid assembly kinetics upon addition of NaCl, a phenomenon also observed recently for the protein A1‐LCD which is the prion‐like LCD of heterogeneous nuclear ribonucleoprotein A1 (hnRNPA1) (Das et al., 2025).

To assess the effect of ionic strength compared with NaCl addition specifically, we measured condensate propensity and amyloid assembly in 170 mM ammonium acetate to match the addition of 150 mM NaCl to 20 mM ammonium acetate (total ionic strength in both cases of 170 mM). We found that the Q331K variant again did not assemble into condensates (Figures S6 and S7), but that amyloid formation still occurred under these elevated ionic strength conditions (Figure S8), akin to the situation observed where amyloid formation could be tuned by addition of NaCl (Figure 4c,d). This suggests that it is unlikely that the effects observed are due to Na^+^ and Cl^−^ ion binding specifically. We also observed that the WT TDP‐43 CTD formed amyloid much faster in 170 mM ammonium acetate than under identical ion strength conditions but in the presence of NaCl (compare Figures 4d and S8), which suggests that different ions may alter amyloid assembly differently. Additionally, we explored the role of condensate assembly by the WT TDP‐43 CTD on the rate of amyloid assembly by adding 5% v/v 1,6‐hexanediol, which is known to dissolve biomolecular condensates (Zheng et al., 2025). It has been observed that in the case of the protein A1‐LCD, biomolecular condensates act as metastable sinks that divert proteins away from forming amyloid because condensate interiors suppress nucleation and fibril growth (Das et al., 2025). Here, we observe a similar effect for TDP‐43 CTD, as when condensate assembly is suppressed by the addition of 5% (v/v) 1,6‐hexanediol the rate of amyloid assembly of the WT TDP‐43 CTD is dramatically increased (Figure S9).

Combined, these data suggest that two independent amyloid assembly routes exist for the TDP‐43 CTD variants: a condensate‐mediated pathway and a salt‐driven pathway that does not require condensate formation, but both involve conformational expansion of the monomeric precursor to initiate each assembly process.

**FIGURE 5:**
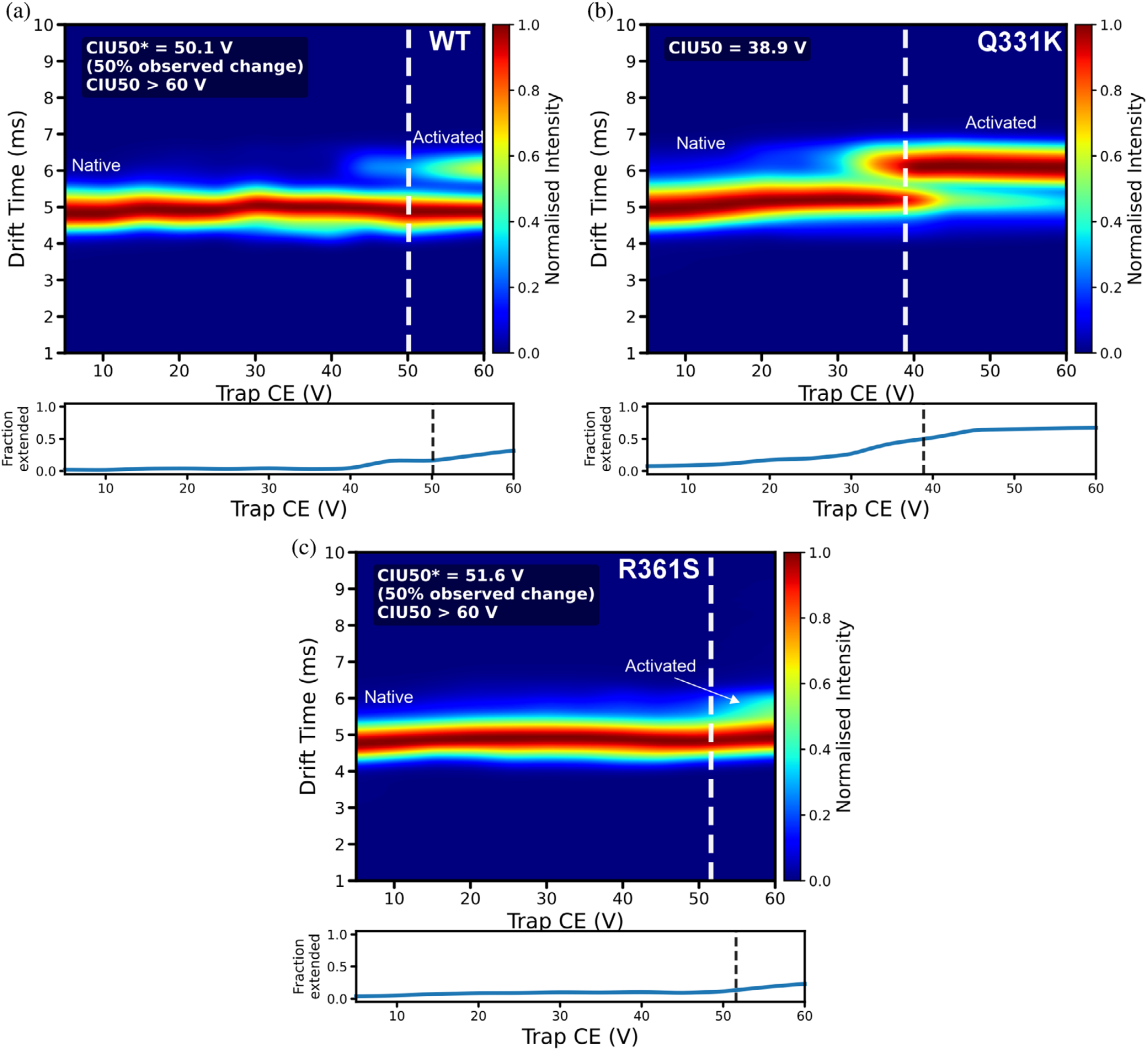
Collision‐induced unfolding (CIU) fingerprints highlight helix‐dependent stability of TAR DNA‐binding protein 43 (TDP‐43) C‐terminal domain (CTD) variants. Normalized heat maps show drift time (ms; *y*‐axis) as a function of trap collision energy (CE in V, *x*‐axis) for the 7+ charge state of the wild‐type, Q331K, and R361S TDP‐43 CTD (see Section 5). Color scale (right; *z*‐axis) denotes signal intensity. Dotted lines indicate the two conformations. The native conformation was restricted to a drift time window of approximately 4.0–5.5 ms and the activated conformation 5.6–7.0 ms for CIU50 and CIU50* calculation. CIU50* represents 50% of the observed change as determined by normalized intensities, plotted as fraction extended below each heat map.

Next, we subjected the 7+ charge state of the three CTD variants from native mass spectrometry to collision‐induced unfolding (CIU; Figure 5) to investigate whether differences in gas phase stabilities between variants could be observed. We chose this charge state because it was an abundant lowly charged species that was present in all the mass spectra we acquired, and therefore these data will reflect unfolding from the most compact state. CIU experiments were performed under low ionic strength conditions (20 mM ammonium acetate) to ensure that the protein remained monomeric and to maintain a stable electrospray. Attempts to perform CIU under elevated ionic strength (150 mM NaCl or 170 mM ammonium acetate) were hindered by rapid phase separation and aggregation over the extended acquisition period required for sequential voltage ramps, preventing reproducible measurement. CIU was therefore only used here to assess intrinsic conformational stability differences between variants in the absence of salt. Previous work has demonstrated that, for folded proteins, the number of unfolding transitions in a CIU experiment is correlated with the number of folded domains, and that the voltage required to elicit unfolding can (relatively) correlate with domain stability (Borotto et al., 2022; Gadkari et al., 2020). The WT CTD was observed to undergo an unfolding transition at ~55 V. Interestingly, the Q331K variant underwent an unfolding transition at markedly lower energy (~30 V). In contrast, the R361S mutant exhibited a much higher stability with evidence of an initial transition occurring at ~60 V. These fingerprints, acquired in the absence of salt, establish a stability order of R361S > WT > Q331K, reflecting the order of in vitro condensation propensity observed by nephelometry (Figure 4). Transition midpoints (CIU50 values) were determined by fixing drift time windows corresponding to native and activated species. For WT and R361S TDP‐43 CTD a full transition to activated species was not observed, and therefore we quantitatively measured CIU50*, which corresponds to 50% of the observed transition (~50 V; Figure 5a,c). To observe a complete transition, a collision voltage greater than 60 V was required; however, this resulted in fragmentation of the proteins and loss of signal. A complete transition for Q331K was observed with a CIU50 of ~40 V (Figure 5b). We interpret these data as evidence that the Q331K substitution perturbs the structural stability of CTH as a result of the mutation to a positive charge. Rather than implying a simple relationship between increased disorder and reduced aggregation, we propose that the CTH may function as a structured interaction element that contributes to productive intermolecular contacts during condensate formation. Destabilization of this region by the Q331K mutation may therefore reduce helix‐mediated interactions required for condensate assembly. Additionally, the introduction of a lysine at position 331 alters local charge patterning, which may independently influence intermolecular association and solubility.

These data demonstrate that CIU can provide a rapid mass‐spectrometric read‐out of how single‐site mutations remodel the conformational landscape of an intrinsically disordered, amyloid‐prone region, even when a global CCS analysis (Figures 2 and 3) does not suggest any gross structural changes are occurring.

### 2.3 Coarse‐grained molecular dynamics simulations capture the altered condensation properties of Q331K TDP‐43 CTD

**FIGURE 6:**
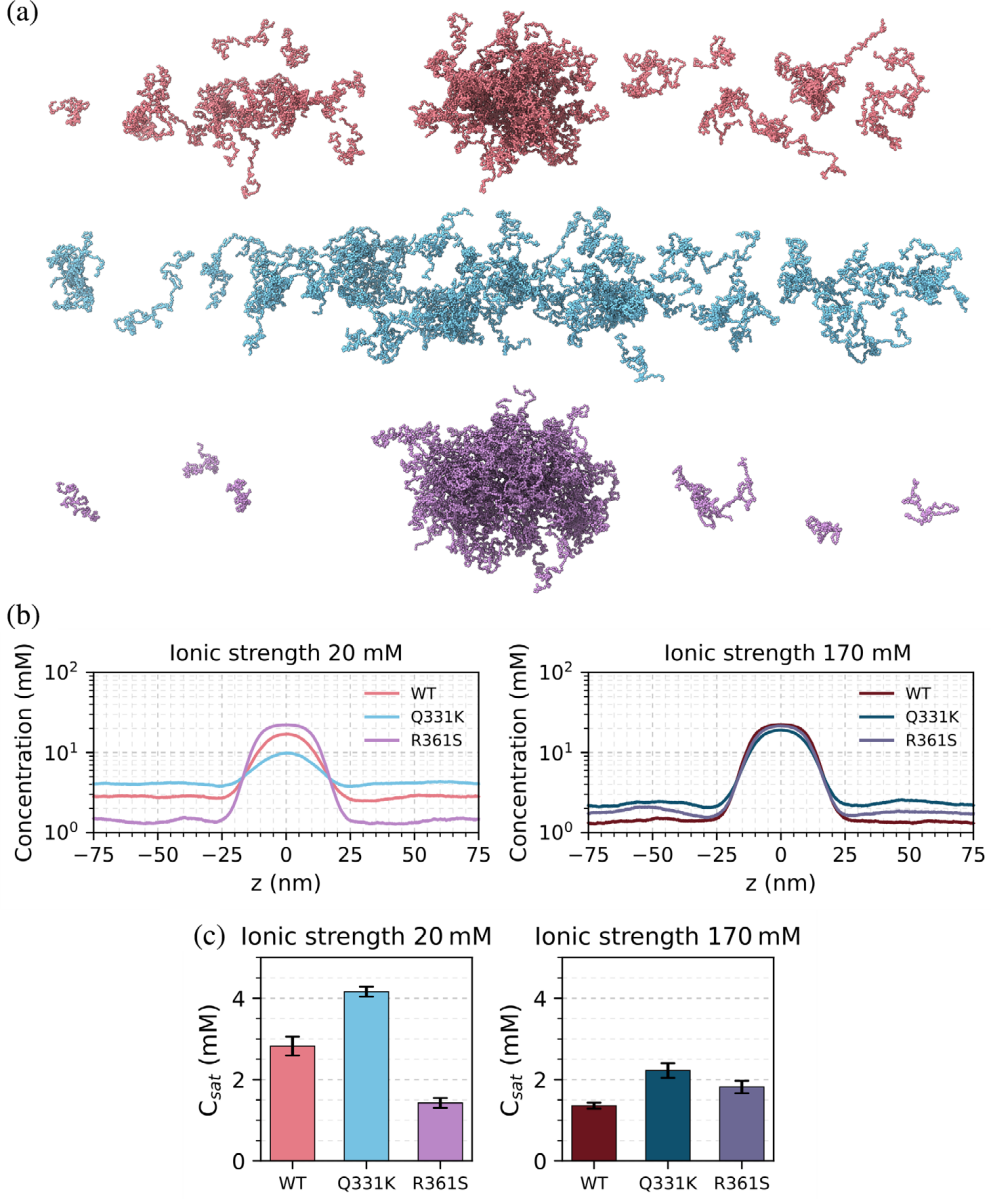
CALVADOS simulations capture salt‐tuned phase separation for the TAR DNA‐binding protein 43 (TDP‐43) C‐terminal domain (CTD). (a) Representative slab snapshots from CALVADOS simulations for wild‐type (WT) (top), Q331K (middle), and R361S (bottom) TDP‐43 CTD. (b) The average concentration of protein across the *z*‐axis of the slab simulation, for simulations performed at low (upper panel) and high (lower panel) ionic strength. (c) Calculation of *C*_sat_ from the region of the simulation outside of the dense phase in the *z*‐axis.

Following our in vitro observations, we applied coarse‐grained molecular dynamics using the CALVADOS2 force field, which applies a one‐bead‐per‐residue model to describe the behavior of multi‐chain conformational ensembles of IDPs, to perform slab simulations of the TDP‐43 CTD and model its phase separation behavior (Cao et al., 2024). To ensure consistency with experimental conditions, CALVADOS2 slab simulations were performed at pH 5.5 and at low ionic strength (20 mM) and high ionic strength (170 mM), matching the IM‐MS and in vitro assays. We note that CALVADOS2 treats the CTD as intrinsically disordered and does not explicitly model the CTH or potential helix–helix packing interactions. Therefore, simulation‐derived conclusions regarding overall condensate propensity and interaction balance within the dense phase are considered robust, whereas specific residue‐residue contact patterns involving the CTH region should be regarded as hypothesis‐generating. Given that the CTH is transient in solution (Conicella et al., 2020) and its structural state within condensates remains unresolved, the simulations are interpreted as probing condensate stability and intermolecular interaction balance after phase separation rather than helix‐driven nucleation events. Nevertheless, in these simulations, the WT and R361S TDP‐43 CTD variants readily formed condensates, producing a single dense phase and a dilute phase (Figure 6a,b). By contrast, the Q331K variant formed condensates to a lesser extent (Figure 6a,b), consistent with our DIC and nephelometry measurements (Figure 4a,b). The one‐dimensional density profiles (Figure 6b) quantify this observation, wherein, at low ionic strength the WT and R361S TDP‐43 variants display clear dense‐ and dilute‐phase plateaus, whereas the profile for the Q331K variant remains at near dilute‐phase levels throughout the simulation window. Elevating the ionic strength increases the dense‐phase concentration and reduces the dilute‐phase concentration for all variants (Figure 6b).

**FIGURE 7:**
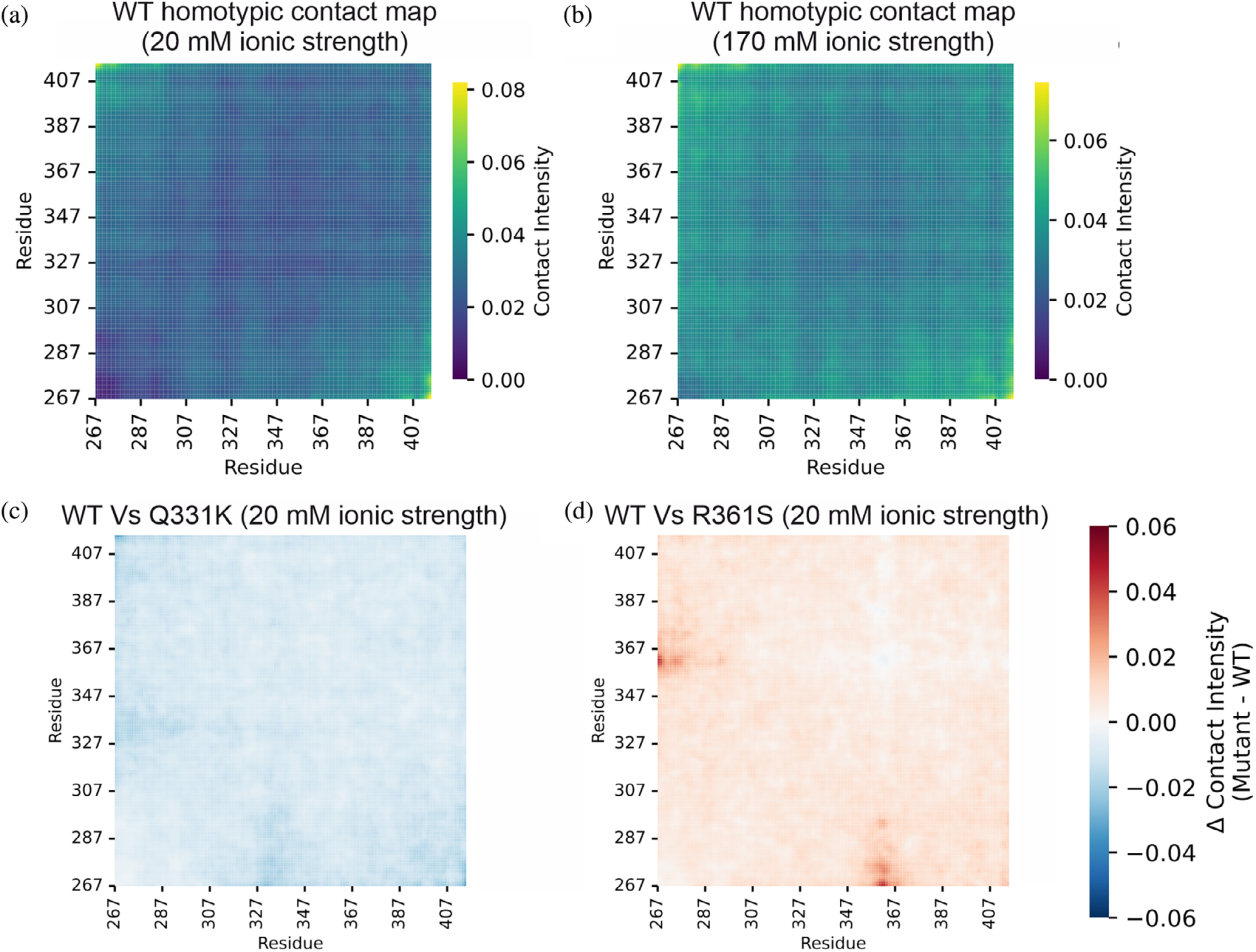
CALVADOS simulations map salt‐tuned contact networks in the TAR DNA‐binding protein 43 (TDP‐43) C‐terminal domain (CTD) and changes in homotypic interactions in TDP‐43 CTD variants. (a, b) Homotypic residue‐residue contact maps for wild‐type (WT) TDP‐43 CTD at (a) 20 mM and (b) 170 mM ionic strength. (c, d) Differential homotypic residue‐residue contact maps in 20 mM ionic strength conditions when comparing (a) Q331K with WT TDP‐43 CTD and (b) R361S TDP‐43 with WT TDP‐43 CTD.

From these data, we can extract a saturation concentration required to yield a condensate core (*C*_sat_; Figure 6c). These *C*_sat_ values mimic the hierarchy of condensate propensity we observed experimentally (R361S > WT > Q331K; Figure 4). We also plotted homotypic contact maps for the WT TDP‐43 CTD at 20 and 170 mM ionic strength, respectively (Figure 7a,b), which are generated by taking the TDP‐43 CTD molecule which is closest to the center of the dense phase in the *z*‐axis of the CALVADOS slab simulation and therefore represents a molecule at the center of the condensate. We then calculated its residue contacts to all other CTD molecules around it. We performed our analysis in this way to exclude any molecules at the edges of the slab. We observe that N‐ and C‐termini interact between molecules under low ionic strength conditions (20 mM; N and C terminal residues have highest contact intensities in Figure 7a) and that intermolecular interactions increase under elevated ionic strength conditions (170 mM), as reflected by higher contact intensities throughout the sequence (Figure 7b). This is consistent with elevated NaCl levels promoting condensate formation. Similar findings were observed for the Q331K and the R361S variants (Figure S10).

Difference contact maps were plotted to compare the contact maps for WT TDP‐43 CTD with those for the Q331K at 20 and 170 mM ionic strength conditions, respectively (Figures 7c and S11) and R361S (Figures 7d and S11) variants. These demonstrate that intermolecular interactions are reduced for the Q331K mutant compared to WT TDP‐43 CTD, with the biggest differences in interactions with the N‐terminus being in residues that include the mutation site (Q327‐M414; Figure S12). We propose that this is because interactions with the positively charged N‐terminus of the chain are reduced because of the introduced Lys residue. These data are consistent with our observation that the Q331K variant of TDP‐43 CTD has reduced propensity to form condensates in vitro (Figure 4). Conversely, intermolecular contacts are increased across the sequence for the R361S variant compared to WT TDP‐43 under low ionic strength (20 mM), with the biggest differences occurring in residues M359‐A366 (Figure S12). This region includes the Ser residue that has been added, and we propose that this substitution promotes interactions with the positive N‐terminus. Whereas, at high ionic strength (170 mM; Figure S11) the differences are reduced suggesting that WT and R361S TDP‐43 CTD behave more similarly under this condition.

In summary, these coarse‐grained molecular dynamics slab simulations using the CALVADOS2 forcefield recapitulated in vitro measurements of condensate formation propensity (Figure 4) demonstrating a hierarchy for condensate propensity of: Q331K < WT < R361S. This observation is consistent with recent benchmarking that demonstrates the model's ability to order relative propensities of IDPs to form biomolecular condensates (Tesei & Lindorff‐Larsen, 2022). However, CALVADOS omits the known α‐helical structure of the CTH and therefore could be underestimating the solubilizing effect of the helix for WT/R361S, while overestimating the condensate formation propensity of Q331K, which introduces an extra positive charge and might prevent dimerization via helix–helix interactions (Conicella et al., 2020).

## 3 DISCUSSION

**FIGURE 8:**
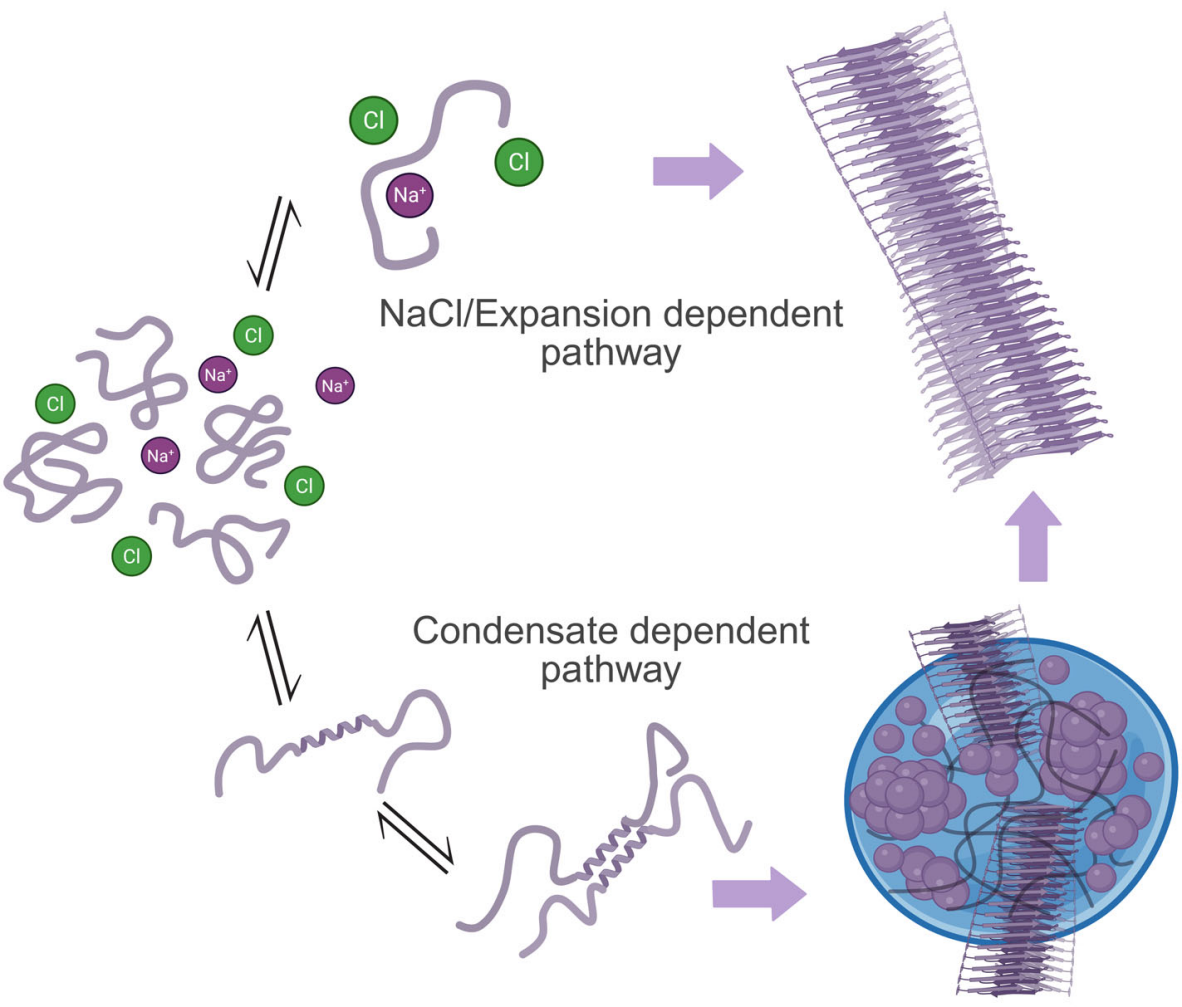
Model of dual amyloid assembly pathways of TAR DNA‐binding protein 43 (TDP‐43) C‐terminal domain (CTD). A schematic depicting two possible routes to amyloid assembly. In the upper pathway, amyloid formation proceeds from solution and is accelerated by the addition of NaCl which increases the abundance of expanded TDP‐43 CTD conformations. In the lower pathway, CTH‐mediated interactions stabilize TDP‐43 CTD condensates and this precedes amyloid assembly. Arrows indicate the direction of structural progression.

It has been reported previously that the CTH within the TDP‐43 CTD is integral for facilitating inter‐protein interactions which stabilize biomolecular condensates (Conicella et al., 2016, 2020). Here, we have explored how a single mutation (Q331K) in the CTH controls the TDP‐43 CTD conformational ensemble, its amyloid fibril formation kinetics and propensity to form biomolecular condensates using an integrative approach. Studying this variant has enabled us to demonstrate that it is possible to uncouple amyloid assembly from biomolecular condensate formation by a single residue substitution in the CTH associated with disease, suggesting that the TDP‐43 CTD can form amyloid fibrils by two mutually exclusive routes (Figure 8).

In this study we employed submicron nanopipette nanoESI emitters which uniquely enable native MS studies in high ionic strength buffers. This builds on our recent work studying the protein α‐synuclein (Byrd, Norgate, et al., 2025), and the work of others who have shown the many benefits of using sub‐micron emitters for native MS (Báez Bolivar et al., 2021; Drachman et al., 2024; Jordan et al., 2022; Panczyk et al., 2020). Here, our native IM‐MS data show that addition of NaCl increases the population of more extended conformations of the TDP‐43 CTD monomer (Figure 2a–d), suggesting that salt perturbs intramolecular interactions. This is consistent with the general principle that electrostatic screening weakens long‐range intramolecular interactions, resulting in expansion of IDP structures/ensembles, therefore priming the protein chain to instead form intermolecular interactions (Das et al., 2025; Garaizar et al., 2020). Although the CTH helix is not explicitly modeled in our coarse‐grained simulations (Figures 6, 7), the results nevertheless recapitulate our in vitro data. While helix formation has been shown to play a role in promoting phase separation (Conicella et al., 2020), our findings suggest that the helical structure may not need to be present in silico to model how mutations alter TDP‐43 CTD condensation propensity.

Strikingly, the Q331K mutation within the CTH abolished condensate formation under both solution conditions used in our amyloid assembly kinetics analyses, whereas the mutation R361S, which lies outside of the CTH, did not (Figure 4). Despite condensate assembly being perturbed by this single point mutation, we show that Q331K TDP‐43 CTD amyloid fibrils still form from bulk solution in the absence of pre‐formed condensates, and that amyloid formation by this route/variant can be accelerated by addition of NaCl (Figure 4c,d). Our native IM‐MS data demonstrate that in the absence of NaCl, monomeric TDP‐43 CTD populates relatively compact conformations, and that added NaCl promotes chain expansion (Figure 3), possibly via electrostatic screening (Chattaraj & Shakhnovich, 2025; Choi & Pappu, 2020; Martin et al., 2020). This suggests that conformational expansion is required to initiate TDP‐43 CTD fibrillation from the monomeric protein in bulk solution. This is consistent with data from single‐molecule FRET and simulations that together have shown that IDPs adopt more expanded conformations in the dense phase of condensates (in a system comprising histone H1 and ProTα) (Galvanetto et al., 2023). We hypothesize that the conformational expansion of TDP‐43 CTD observed in condensates is akin to the conformational expansion occurring in bulk for the Q331K variant upon NaCl addition, and both act to facilitate amyloid assembly via different mechanisms/material states.

Despite our observation of chain extension under elevated salt conditions in our IM‐MS experiments (Figure 2c,d), the WT CTD formed amyloid more slowly than at lower ionic strength, as measured by the length of the lag phase (Figure 4d). We interpret this observation as evidence of the competition between pathways where this promotes partitioning of monomers into condensates thereby depleting amyloid‐competent species from the bulk. Interestingly, when 1,6‐hexanediol, known to dissolve condensates by weakening hydrophobic interactions, was added at 5% (v/v) under high salt concentrations, ThT positive amyloid assembly was accelerated for the WT CTD (Figure S9). Under these conditions, the effective bulk concentration of amyloid‐competent precursors presumably is increased, removing the kinetic “sink” imposed by condensates (Das et al., 2025). Together, these data support a model involving competing mechanisms toward amyloid assembly in which for the WT and R361S TDP‐43 CTD variants, elevated salt favors a condensate‐dominant route that is kinetically slower in amyloid formation, whereas dissolution of condensates redirects the system to the salt‐expanded, fibril forming pathway (Figure 8) (Das et al., 2025). This model is consistent with the data we have obtained for the Q331K TDP‐43 CTD variant, as in the absence of added NaCl amyloid assembly is dramatically retarded, and our IM‐MS is consistent with compact conformations predominating. However, increasing the NaCl levels accelerates amyloid assembly and promotes a conformational expansion of the protein, without triggering condensate formation.

Transient secondary structural elements are known to act as “stickers” that participate in intermolecular interactions to form condensates. In TDP‐43, residues approximately 320–343 form the metastable CTH that mediates helix–helix contacts and contacts with glycine/serine‐rich regions (Chang et al., 2020; Rizuan et al., 2025; Shenoy et al., 2023). Multiple studies have shown that enhancing helical propensity in this region increases the propensity for condensate formation, whereas helix‐breaking substitutions reduce it (Conicella et al., 2016, 2020). Residue Q331 lies within the CTH. The Lys substitution at residue 331 (Q331K) perturbs both charge patterning and helical stability: it introduces a local positive charge, alters the helix macrodipole/side‐chain packing, and disrupts helix–helix pairing (Conicella et al., 2016, 2020; Rizuan et al., 2025). Our findings align with prior reports that mutations within CTH tune condensate formation, highlighting the CTH as a molecular switch that gates access to facilitate condensate assembly (Conicella et al., 2016). Here, we extend this further by revealing how such alterations also tune amyloidogenicity.

Much is known about the complex interplay between amyloid assembly and condensate formation. Condensate assembly can increase local concentration in the dense phase by 10–1000*x* (Garcia‐Cabau & Salvatella, 2021; Tibble & Gross, 2023), yet the dense phase is known to sequester monomers, slowing fibril assembly dependent on protein sequence, salt, pH, and RNA presence (Das et al., 2025; Lipiński et al., 2022). Given that our IM‐MS measurements of WT and R361S TDP‐43 were taken under conditions where condensates were present, it is not possible for us to determine if the protein signal detected corresponds to monomer present in the bulk phase surrounding the condensates or protein ejected from condensates during ionization.

While our IM‐MS derived CCSD analysis of the three TDP‐43 variants revealed they adopted broadly similar structural ensembles (Figures 2 and 3), CIU measurements did reveal stability differences (Figure 5). Given the correlation of the number of CIU unfolding events with structured domains in proteins, the CIU fingerprints (Figure 5) could represent unfolding of the CTH but also unfolding via disruption of long‐range intramolecular interactions which stabilize the over‐compact protein conformation of TDP‐43 CTD (Figures 2 and 3). Interestingly, the Q331K variant presented a much less stable CIU fingerprint (for the 7+ charge state) than the WT protein (Figure 5), which might reflect weaker intramolecular interactions formed when a positive charge is added at this site in the CTH, which is also reflected in our protein–protein interaction maps (Figure 7). This demonstrates that by combining global CCSD analysis with CIU experiments, it is possible to unpick subtle conformational changes in disordered proteins using native MS.

It is important to note that the situation in vivo/in cell is much more complex than our in vitro experiments, with co‐condensation known to play pivotal roles in cellular condensate assembly (Zhang et al., 2024). Molecular chaperones such as HSP70 and HSP40 (Gu et al., 2020; Li et al., 2022) and other client biomolecules such as RNA can partition into condensates where they can bind to sticker motifs on proteins undergoing condensate assembly to increase fluidity of the dense phase. HSP70 has been shown to partition into condensates and chaperone condensed fused in sarcoma (FUS) protein to prevent amyloid conversion, and when HSP70 is depleted, condensates undergo a liquid‐to‐solid transition, and amyloid assembly ensues (Li et al., 2022). Additionally, in a cellular context, as well as the assembly of multicomponent condensates, PTMs, crowding and ionic composition may also play a role in deciding amyloid assembly pathways. Combined, these factors add to the complexity that must be explored if we are to fully understand the mechanisms that govern condensate mediated amyloid assembly. While bulk cytosolic ionic strength and pH are tightly regulated under homeostasis, cellular stress (oxidative stress, osmotic challenge, or stress granule formation) can locally alter ion strength, protonation states, and electrostatic screening. These changes can modulate the balance between intramolecular contacts and intermolecular interactions, thereby shifting TDP‐43 from a condensate‐mediated assembly route toward a salt‐expanded amyloid pathway under conditions that mimic the altered physicochemical environments associated with stress responses.

Although our data support electrostatic screening as a mechanism by which ionic strength modulates CTD expansion and condensate assembly, we have not systematically explored ion‐specific effects, including Hofmeister ordering and multivalent cations. Future work systematically varying ion identity and valency will therefore be essential to distinguish purely electrostatic screening effects from ion‐specific modulation of TDP‐43 condensate assembly and fibrillation.

## 4 CONCLUSION

Our integrative analysis combining native mass spectrometry, biochemical characterization supported by coarse‐grained simulations identifies the sequence of the CTH of TDP‐43 as the decisive element that couples condensate formation with amyloid assembly. This mechanistic framework reveals competing self‐assembly pathways toward amyloid assembly and suggests that understanding the molecular mechanisms that govern the choice between alternative self‐assembly pathways might be crucial for elucidating disease mechanisms.

## 5 METHODS

### 5.1 Recombinant TDP‐43 CTD expression and purification

A plasmid containing the DNA sequence for the CTD of TDP‐43 (residues 267–414) was kindly gifted from Nicolas Fawzi (Addgene plasmid #98669) and mutations Q331K and R361S were introduced by site‐directed mutagenesis (Q5® site‐directed mutagenesis kit, New England Biolabs). For protein expression, chemically competent BL21 DE3 *Escherichia coli* cells were transformed with plasmids containing the TDP‐43 CTD gene. Cells were grown in Luria broth (LB) media with kanamycin (50 μg/mL) at 37°C with shaking (200 rpm) to an OD_600_ of 0.6, and protein expression was induced by the addition of 0.01 mg/mL isopropyl β‐D‐1‐thiogalactopyranoside (IPTG) for 4 h at 37°C, 200 rpm. Cells were harvested by centrifugation, resuspended in lysis buffer (20 mM Tris‐Cl, 300 mM NaCl, 10 mM imidazole, 1 mM dithiothreitol (DTT), pH 8.0) supplemented with an ethylenediamine tetraacetic acid (EDTA)‐free protease inhibitor cocktail (Roche) and lysed using a cell disruptor (Constant Cell Disruption Systems). The lysate was centrifuged (20,000 × *g*, 60 min) and the insoluble material, including inclusion bodies (IBs) containing the expressed TDP‐43 CTD, was resuspended in denaturing binding buffer (20 mM Tris‐Cl, 300 mM NaCl, 10 mM imidazole, 1 mM DTT, 8 M urea, pH 8.0). The solubilized IBs were centrifuged (20,000 × *g*, 60 min) to remove remaining insoluble debris and then applied to a 5 mL Histrap HP column (Cytiva). The column was washed with five column volumes of binding buffer, before elution with a linear gradient (0%–100% B) of buffer B (20 mM Tris‐Cl, 300 mM NaCl, 500 mM imidazole, 1 mM DTT, 8 M urea, pH 8.0) over 20 column volumes. For His‐tag cleavage, the protein was incubated with Tobacco Etch Virus (TEV) (1:25 w/w) protease at 4°C overnight while dialyzing into 20 mM 2‐(N‐morpholino)ethanesulfonic acid (MES) buffer, pH 5.5. Cleaved protein was resuspended in denaturing binding buffer and reapplied to a 5 mL Histrap HP column (Cytiva). The flow through was collected and concentrated to ~1 mM using Amicon® 10 kDa ultra centrifugal filter units (Merck Millipore, Darmstadt, Germany) before being snap‐frozen and stored at –80°C. Aliquots were stored at high protein concentrations (~1 mM) in 8 M urea to prevent aggregation and condensate assembly, and were diluted into 20 mM ammonium acetate, pH 5.5 immediately prior to analyses. Analyses were carried out at pH 5.5 to maintain protein solubility, consistent with previous studies (Dang et al., 2021; Pakravan et al., 2021).

### 5.2 Nanopipette nanoESI emitter fabrication

The nanopipette nanoESI emitter tips were fabricated using 1.0 mm outer diameter and 0.5 mm inner diameter quartz capillaries (QF100‐50‐7.5; Sutter Instrument) using the Sutter Instrument P2000 laser puller (World Precision Instruments). A two‐line protocol was used: line 1 with HEAT 750/FIL 4/VEL 30/DEL 150/PUL 80, followed by line 2 with HEAT 850/FIL 3/VEL 40/DEL 135/PUL 225. This protocol generates nanopipettes with openings of ~40 nm in diameter (Byrd, Norgate, et al., 2025). The pulling protocol is specific to the instrument and can vary between different pullers, so individual optimization of the protocol is needed in each laboratory. For native MS, emitters were filled with analyte solution and fitted with a platinum wire (PT00‐WR‐000117; Goodfellow) prior to use.

### 5.3 Native ion mobility‐mass spectrometry

Native IM‐MS experiments were performed on a Synapt G2‐Si HDMS mass spectrometer (Waters Corporation, Wilmslow, UK) with traveling (T‐wave) ion mobility and a nanoESI source. TDP‐43 CTD variants (WT, Q331K, R361S) were analyzed at a concentration of 10 μM in 20 mM ammonium acetate (Byrd et al., 2023; Byrd, Norgate, et al., 2025; Byrd, Rowlinson, et al., 2025), pH 5.5. Instrument parameters were as follows: capillary voltage 1–1.4 kV, source temperature 30°C, sampling cone 18 V, trap collision energy 5 V, transfer collision energy 2.0 V, trap direct current (DC) bias 30 V, IM wave velocity 550 m/s, IM wave height 10 V. The trap gas flow was 2.0 mL/min, ion mobility gas flow was 6.0 mL/min (using N_2_ gas) and the helium gas flow was 80.0 mL/min. The IM spectra were calibrated (Bush et al., 2010) using denatured cytochrome c (charge states 13+ to 19+), myoglobin (charge states 15+ to 24+) and ubiquitin (charge states 7+ to 13+), 10 μM solutions of each calibrant in 50% (v/v) acetonitrile, 0.1% (v/v) formic acid were used for calibration to obtain CCSDs for each TDP‐43 CTD charge state detected. Here we term these values ^TW^CCS_N2_, consistent with community standards (Bush et al., 2010), signifying that the CCS values were calculated using traveling wave ion mobility in N_2_ buffer gas using calibrants acquired in N_2_ buffer gas. Note that for calibration of our traveling wave IM data, we have used denatured protein standards. Our rationale for this choice is that TDP‐43 is an IDP and therefore unfolded/denatured proteins could be more reflective of the structural properties of the protein compared with natively folded proteins (Cheung See Kit et al., 2023; Christofi & Barran, 2023; Jeacock et al., 2023; Moons et al., 2020). It is important to note that calibrant choice can influence the calibrated CCS values obtained from traveling wave IM experiments, and we have carefully chosen appropriate calibrants that span the *m*/*z* and drift time values of the analytes investigated in this work (Bush et al., 2010, 2012). MassLynx v4.1 (Waters Corporation) was used for data processing. For collision induced unfolding (CIU), instrument parameters were identical except for the trap collision energy which was systematically increased from 5 to 60 V in 5 V increments. Arrival time distributions for the 7+ charge state were selected to generate CIU plots. Drift time profiles were extracted at each collision voltage from the spectral peak using the full width of the peak at half maximum (FWHM) intensity.

### 5.4 ThT amyloid assembly kinetics

Kinetics of TDP‐43 CTD amyloid formation were monitored in a 96‐well, non‐binding, flat‐bottom microplate (Corning; 10438082). Samples (100 μL) containing 50 μM protein with 20 μM ThT in 20 mM ammonium acetate, pH 5.5 with 0 mM NaCl and 150 mM NaCl were incubated at room temperature, quiescently in a FLUOstar Omega plate reader (BMG Labtech). Fluorescence intensity was measured by exciting at 440 ± 10 nm and collecting emission at 482 ± 12 nm using a bandpass filter. Four replicate measurements were conducted, and results were blank corrected and normalized to the maximum fluorescence value of each curve except for when amyloid formation did not occur (in the case of Q331K TDP‐43 CTD variant in the absence of added NaCl) where the curve was normalized to the maximum intensity measured for WT TDP‐43 CTD in the absence of salt.

### 5.5 Negative stain transmission electron microscopy

A sample of 5 μL was taken from the ThT plate at the endpoint of each reaction, loaded onto a glow discharged (30 s, Pelco Easi‐glow), 400 mesh continuous carbon grid, and incubated for 2 min. The sample was blotted and washed twice with H_2_O before being blotted and stained twice with 2% (w/v) uranyl acetate. Grids were imaged on FEI Tecnai T12 electron microscope using a nominal magnification of 30,000×.

### 5.6 DIC microscopy

One hundred microliters of the TDP‐43 CTD (WT, Q331K or R361S) protein was added at a concentration of 50 μM, in 20 mM ammonium acetate, pH 5.5 containing 0 or 150 mM NaCl to individual wells of an 18‐well high glass bottom chamber slide (Ibidi). To generate phase diagrams, 10, 50, 100, and 200 μM were imaged at 0, 50, and 150 mM NaCl in 20 mM ammonium acetate, pH 5.5. Condensates were imaged using a LSM700 Airyscan confocal microscope (Zeiss) using a DIC20× 0.3 objective.

### 5.7 Nephelometry

WT, Q331K and R361S CTD variants were added to a 96‐well, non‐binding, flat‐bottom, half‐area microplate (Corning, USA; 10629151) at a concentration of 50 μM in 20 mM ammonium acetate, pH 5.5 with 0 mM NaCl or 150 mM NaCl added. Light scattering of 40 μL of each solution was then monitored using a Nephelostar plate reader (BMG Labtech, Ortenburg, Germany) using an excitation wavelength of 635 ± 10 nm, over 6 h at 25°C. Data were blank corrected and three replicate measurements were conducted. Significance was assessed using an unpaired two‐sided Welch's *t*‐test on the blank corrected (linear) relative nephelometry unit (RNU) values to account for unequal variances between conditions. Statistical analyses were performed in Python (SciPy). A threshold of *p* < 0.05 was considered significant. Where indicated in figures ** = *p* < 0.01.

### 5.8 Coarse‐grained molecular dynamics simulations

Coarse‐grained implicit‐solvent simulations of the TDP‐43 CTD were performed in the OpenMM framework (version 8.1.1) (Eastman et al., 2017) using the CALVADOS python package and the CALVADOS2 force field (Tesei et al., 2021; Tesei & Lindorff‐Larsen, 2022). Simulations were performed for TDP‐43 CTD WT, Q331K and R361S at low and high ionic strengths (20 and 170 mM). Unless stated otherwise simulation parameters used were kept as their default values for slab simulations in the software. Simulations were performed in a 15 × 15 × 150 nm box with 100 TDP‐43 CTD molecules (pH 5.5). Following energy minimization, simulations were run at a temperature of 21°C for a total of 10 μs each with coordinates being saved every 0.1 ns. The simulation had a time step of 10 fs and a friction coefficient of 10 fs^−1^. The first 0.1 μs of each simulation was considered to still be part of the equilibration time and therefore not included in the analysis. To reduce the total number of frames in the analysis, every 10th frame was included such that the analysis was performed on frame each 1 ns. The *C*_sat_ was calculated with inbuilt functions in the CALVADOS software, where the concentration is calculated for frames with *z* values greater than = ~50 nm and less than approximately −50 nm (i.e., outside the dense phase). For each frame, the central chain was selected as the one closest to the mid‐plane (*z* = 0 nm) of the protein‐dense slab to avoid molecules near the slab interface, as is standard in analyses using the CALVADOS software package.

## AUTHOR CONTRIBUTIONS

**Emily J. Byrd**: conceptualization, methodology, validation, formal analysis, investigation, data curation, writing—original draft, visualization; **Joel A. Crossley**: formal analysis, investigation, data curation, visualization; **Chalmers C. C. Chau**: methodology, resources; **Paolo Actis**: conceptualization, methodology, validation, writing—review and editing, supervision, project administration; **Antonio N. Calabrese**: conceptualization, methodology, validation, writing—review and editing, supervision, project administration, funding acquisition.

## CONFLICT OF INTEREST STATEMENT

None of the authors have a conflict of interest to disclose.

## Supporting information


**Data S1.** Supporting Information.

## Data Availability

The data that support the findings of this study are openly available in University of Leeds Data Repository at https://doi.org/10.5518/1785.
