# Supplementary material for: An ALS‐associated mutation in the C‐terminal α‐helix of TDP‐43 uncouples condensate formation and amyloid assembly
Source: Protein Sci. 2026 Apr 13;35(5):e70565. doi: 10.1002/pro.70565 (PMC13071763; doi:10.1002/pro.70565)
Supplement: Supplementary file 1 — Data S1. Supporting Information. [file PRO-35-e70565-s001.docx]

**Supplementary Information**

**An ALS-Associated Mutation in the C-terminal α-helix of TDP-43 Uncouples Condensate Formation and Amyloid Assembly**

Emily J. Byrd^1^, Joel A. Crossley^1^, Chalmers C. C. Chau^2,3^, Paolo Actis^2,3^, Antonio N. Calabrese^1*^

^1^Astbury Centre for Structural Molecular Biology, School of Molecular and Cellular Biology, Faculty of Biological Sciences, University of Leeds, Leeds, LS2 9JT, UK.

^2^School of Electronic and Electrical Engineering, University of Leeds, LS2 9JT, UK.

^3^Bragg Centre for Materials Research, University of Leeds, LS2 9JT, UK.

* To whom correspondence should be addressed: A.Calabrese@leeds.ac.uk

**
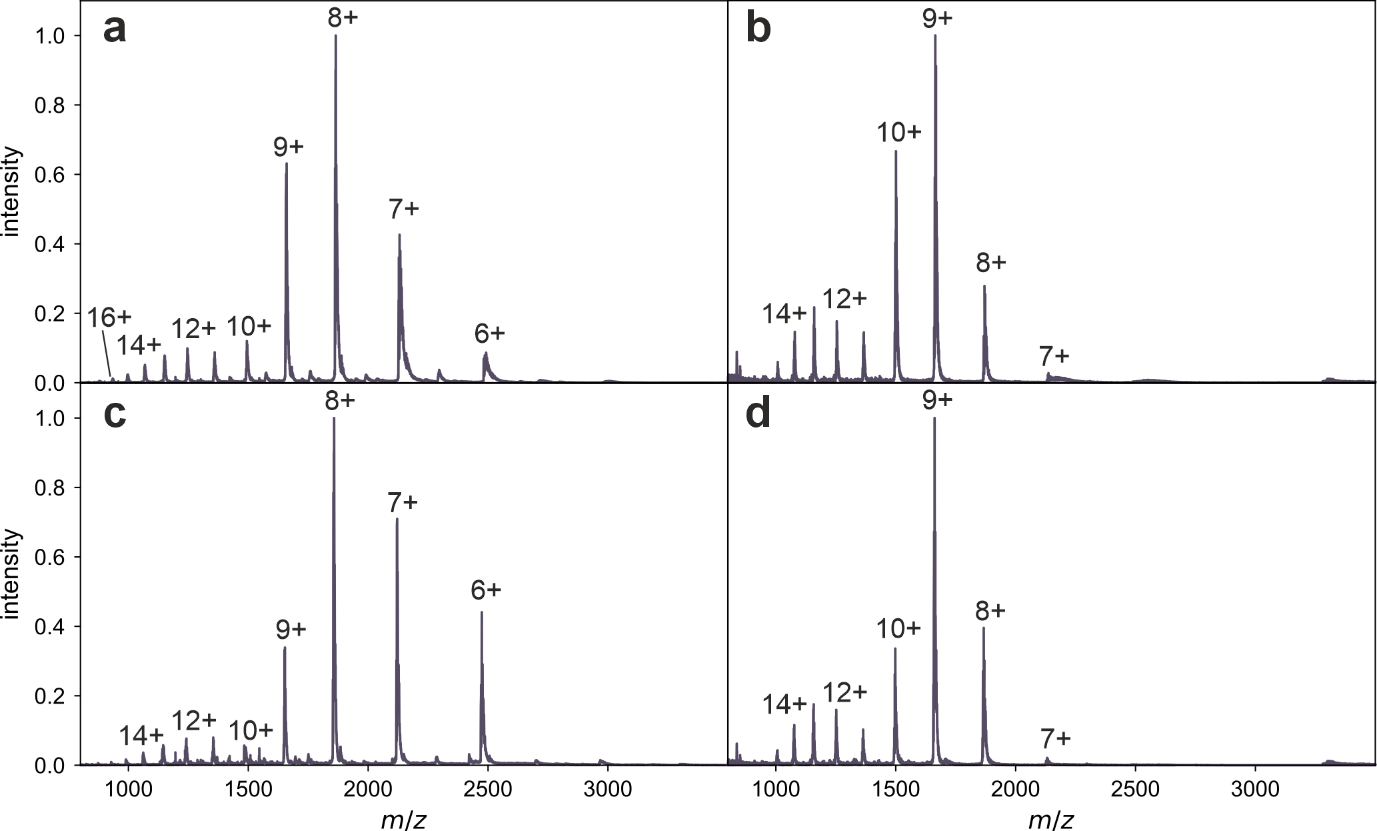
**

***Figure S1.* *Native nanoESI mass spectra of Q331K TDP-43 CTD and R361S TDP-43 CTD under low and high salt conditions.*** *(a) Mass spectrum of Q331K TDP-43 CTD acquired from 20 mM ammonium acetate (pH 5.5). (b) Mass spectrum of Q331K TDP-43 CTD acquired from 20 mM ammonium acetate, 150 mM NaCl (pH 5.5). (c) Mass spectrum of R361S TDP-43 CTD acquired from 20 mM ammonium acetate (pH 5.5). (d) Mass spectrum of R361S TDP-43 CTD acquired from 20 mM ammonium acetate, 150 mM NaCl (pH 5.5). (b) and (d) were acquired using submicron nanopipette nanoESI*^58^*.*

*
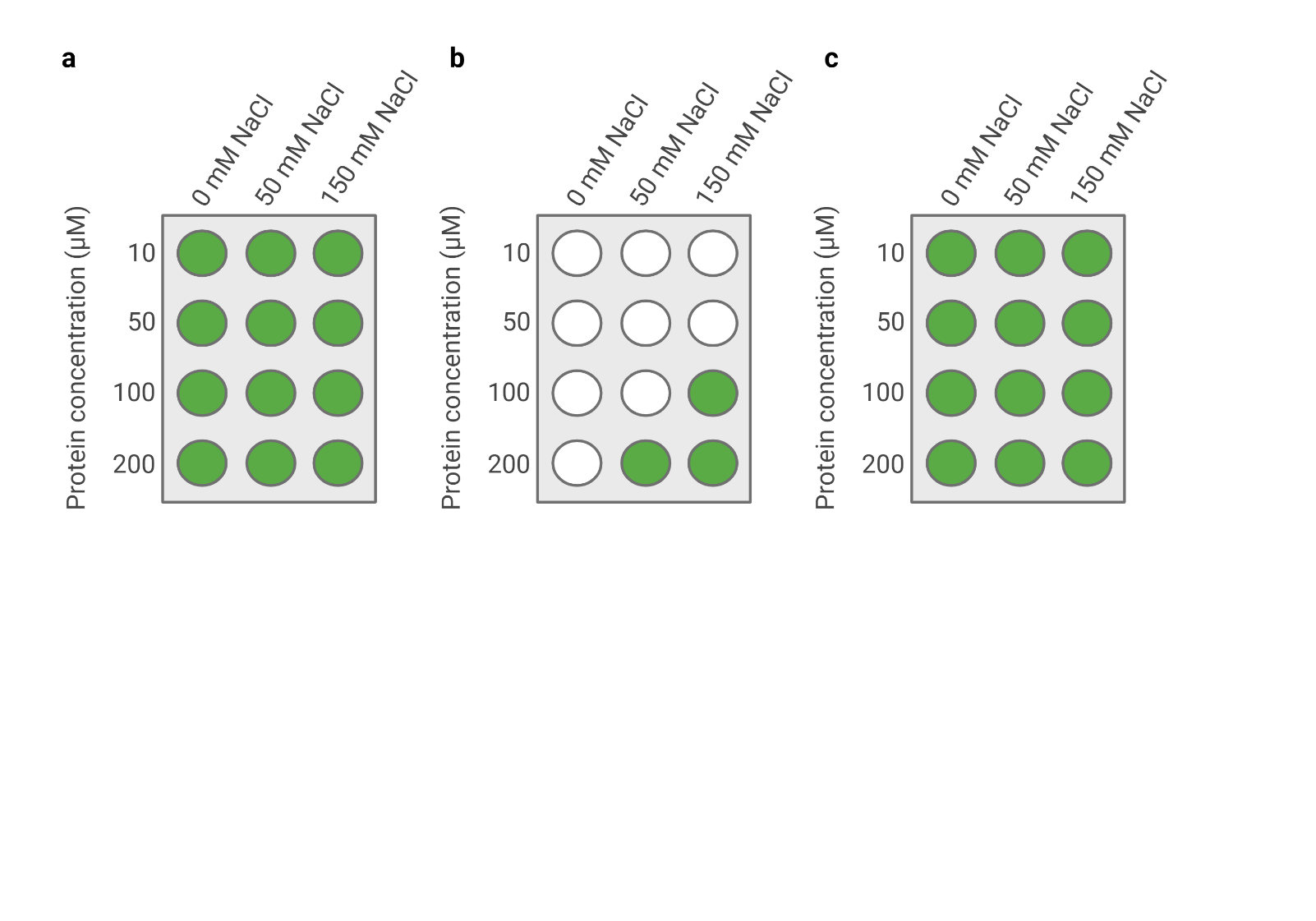
****Figure S2. Phase diagrams of condensate formation by TDP-43 CTD variants.*** *Using DIC microscopy, the phase propensity of the WT TDP-43 CTD (a) the Q331K mutant (b) and the R361S mutant (c) were explored at increasing concentrations of protein (y-axis; 10 µM to 200 µM) and NaCl (x-axis; 0 mM to 150 mM). Green circles indicate conditions where condensates were observed.*

***Figure S3. DIC microscopy of WT TDP-43 CTD.*** *The phase propensity of WT TDP-43 CTD was explored at increasing protein concentration (y-axis) and increasing NaCl concentration (x-axis). Green borders indicate if condensates were observed
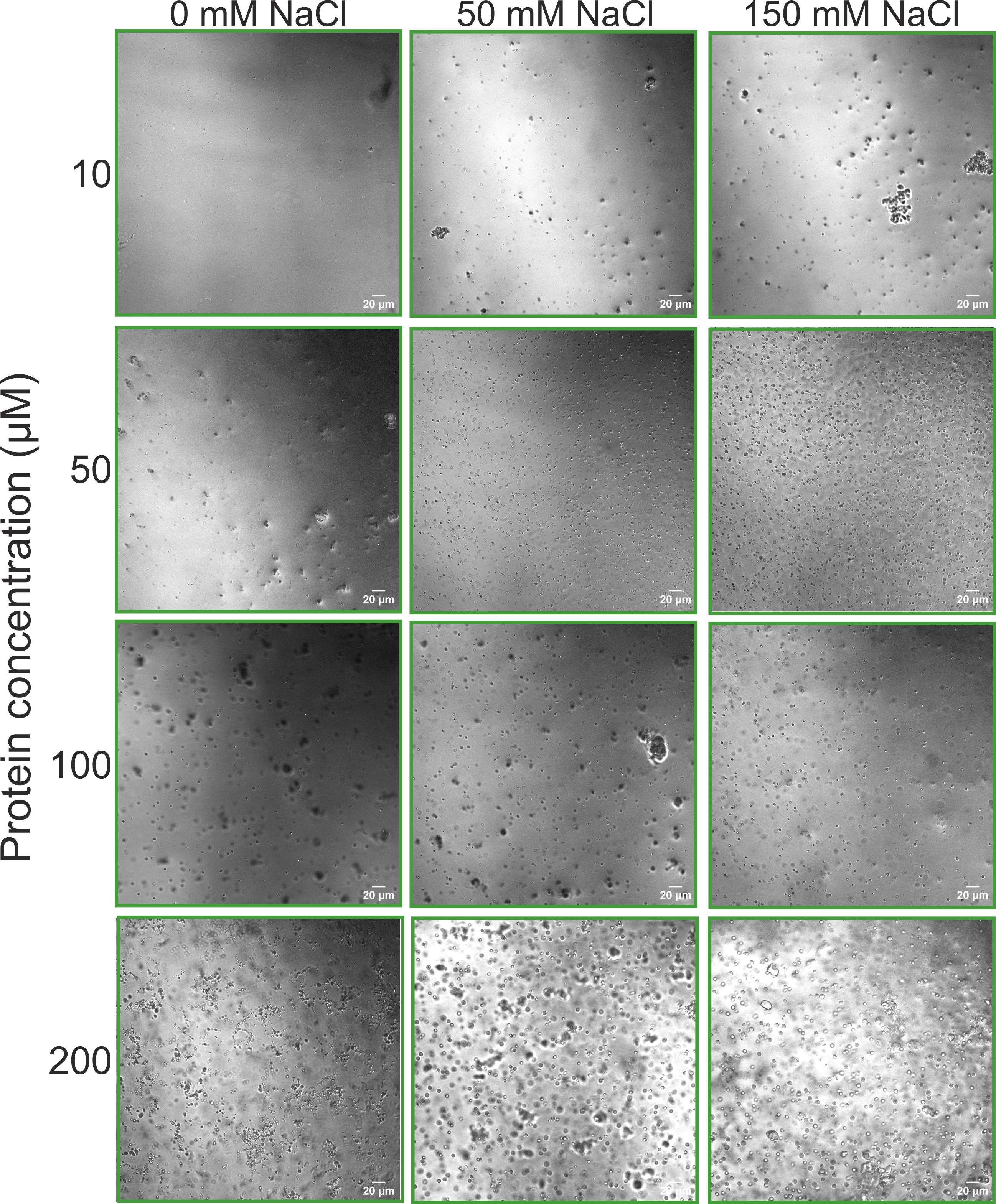
*

***
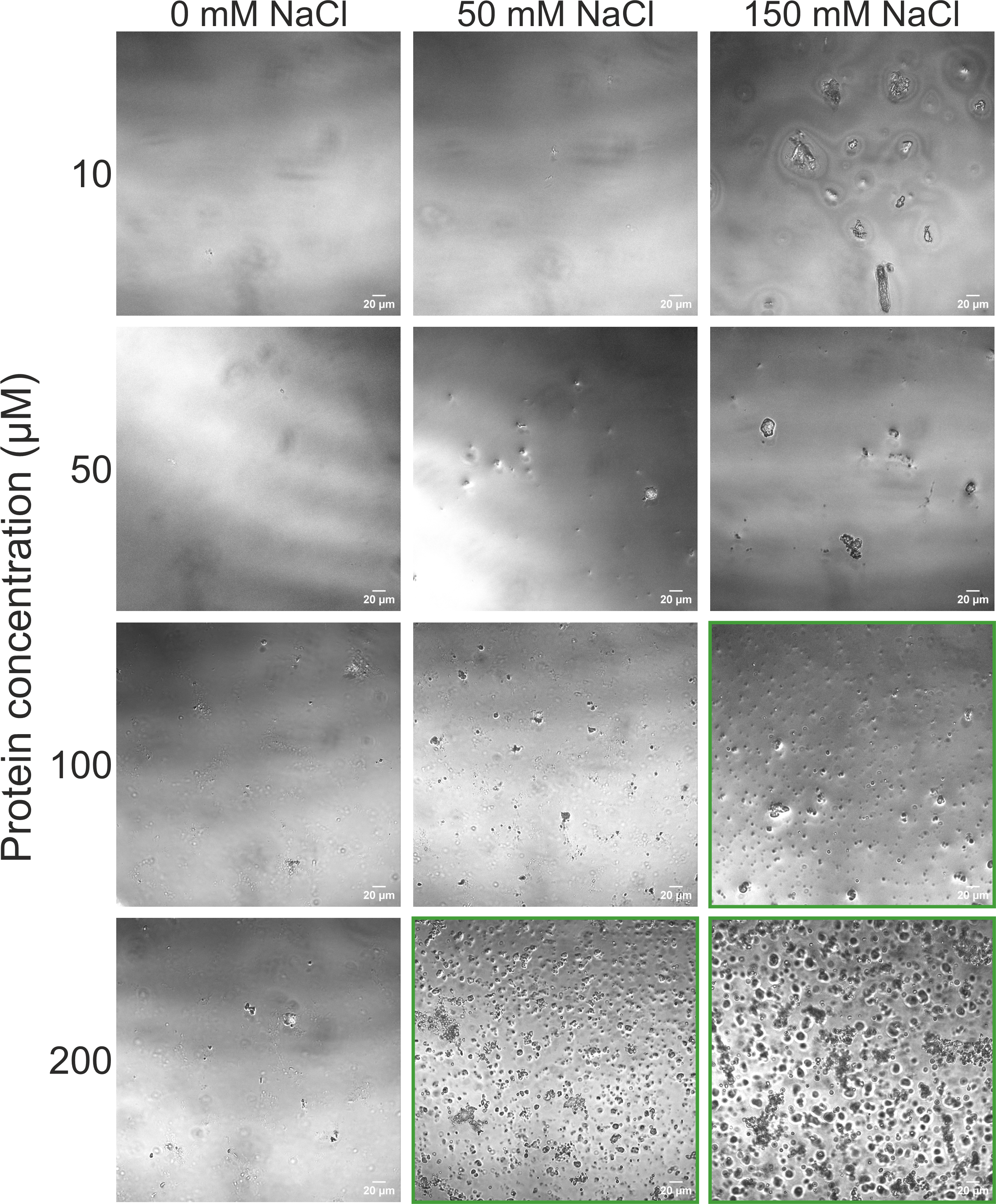
Figure S4. DIC microscopy of Q331K TDP-43 CTD.*** *The phase propensity of Q331K TDP-43 CTD was explored at increasing protein concentration (y-axis) and increasing NaCl concentration (x-axis). Green borders indicate if condensates were observed.*

*
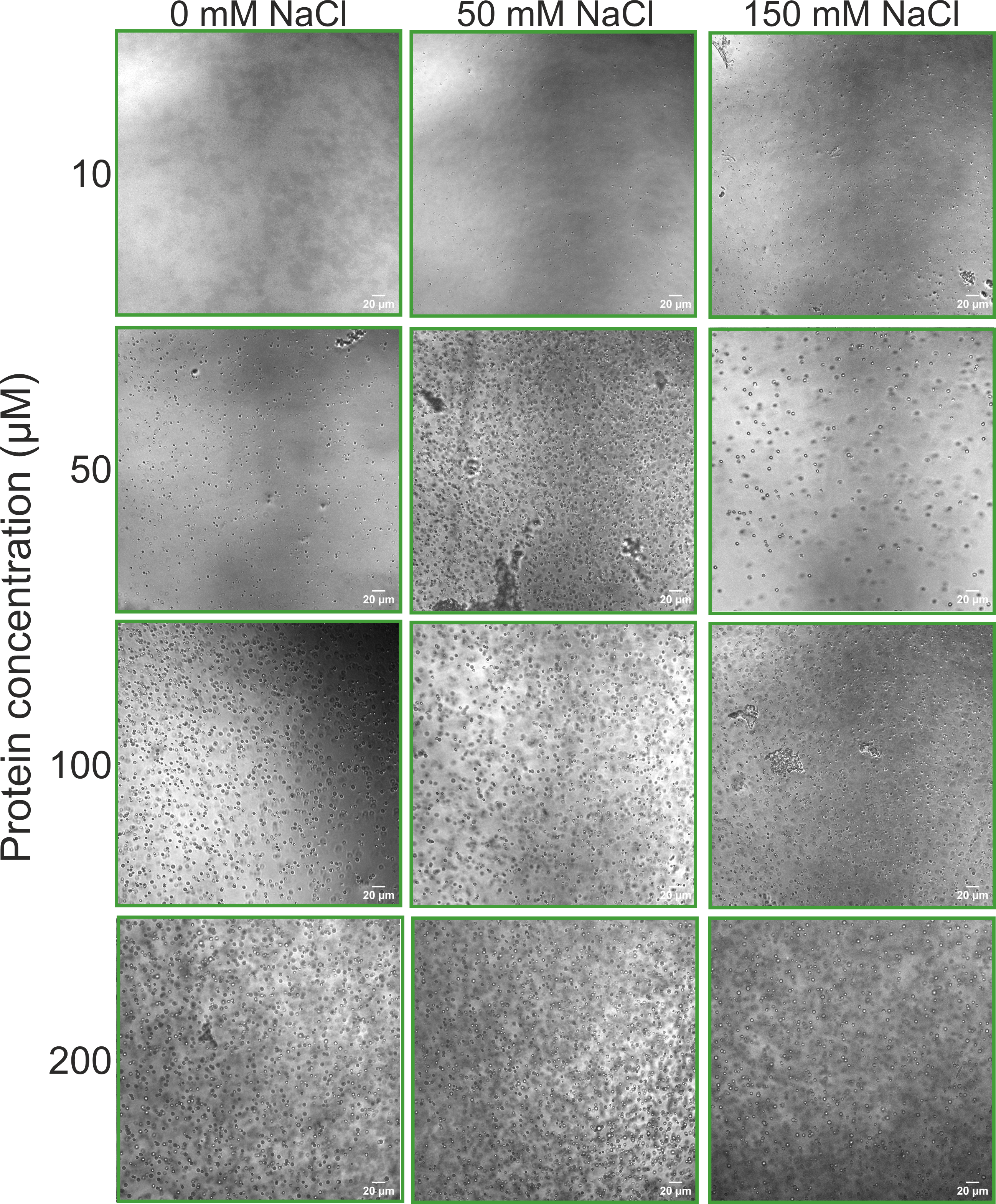
****Figure S5. DIC microscopy of R361S TDP-43 CTD.*** *The phase propensity of the R361S TDP-43 CTD was explored at increasing protein concentration (y-axis) and increasing NaCl concentration (x-axis). Green borders indicate if condensates were observed.*

**
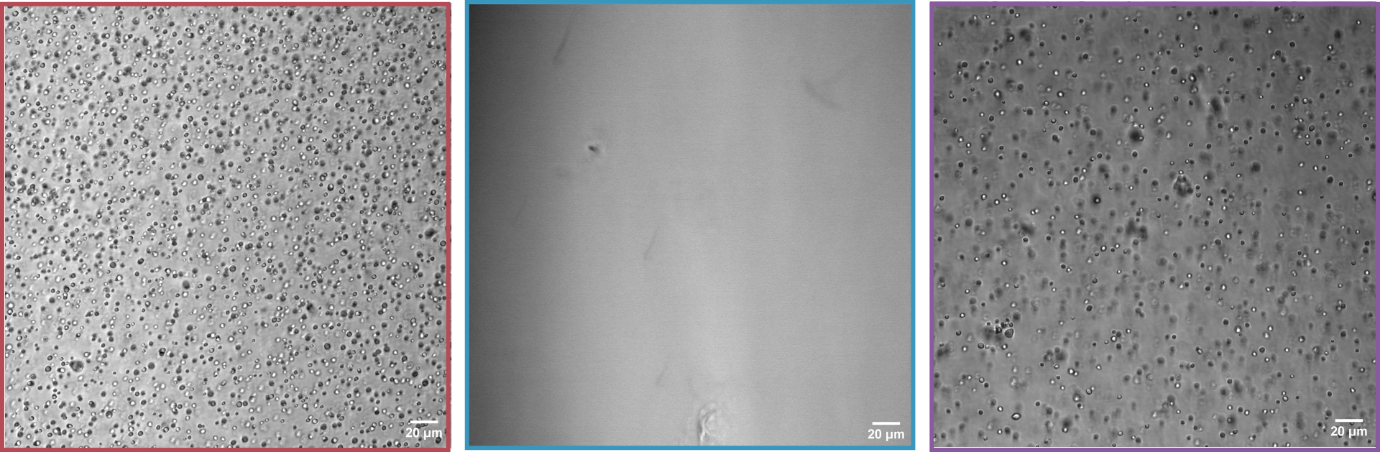
**

***Figure S6. Differential-interference contrast (DIC) microscopy of TDP-43 CTD variants in 170 mM ammonium acetate.*** *DIC microscopy images of solutions containing WT TDP-43 CTD (red, left), Q331K TDP-43 CTD (blue, middle), and R361S TDP-43 CTD (purple, right). Scale bars are 20 µm.*


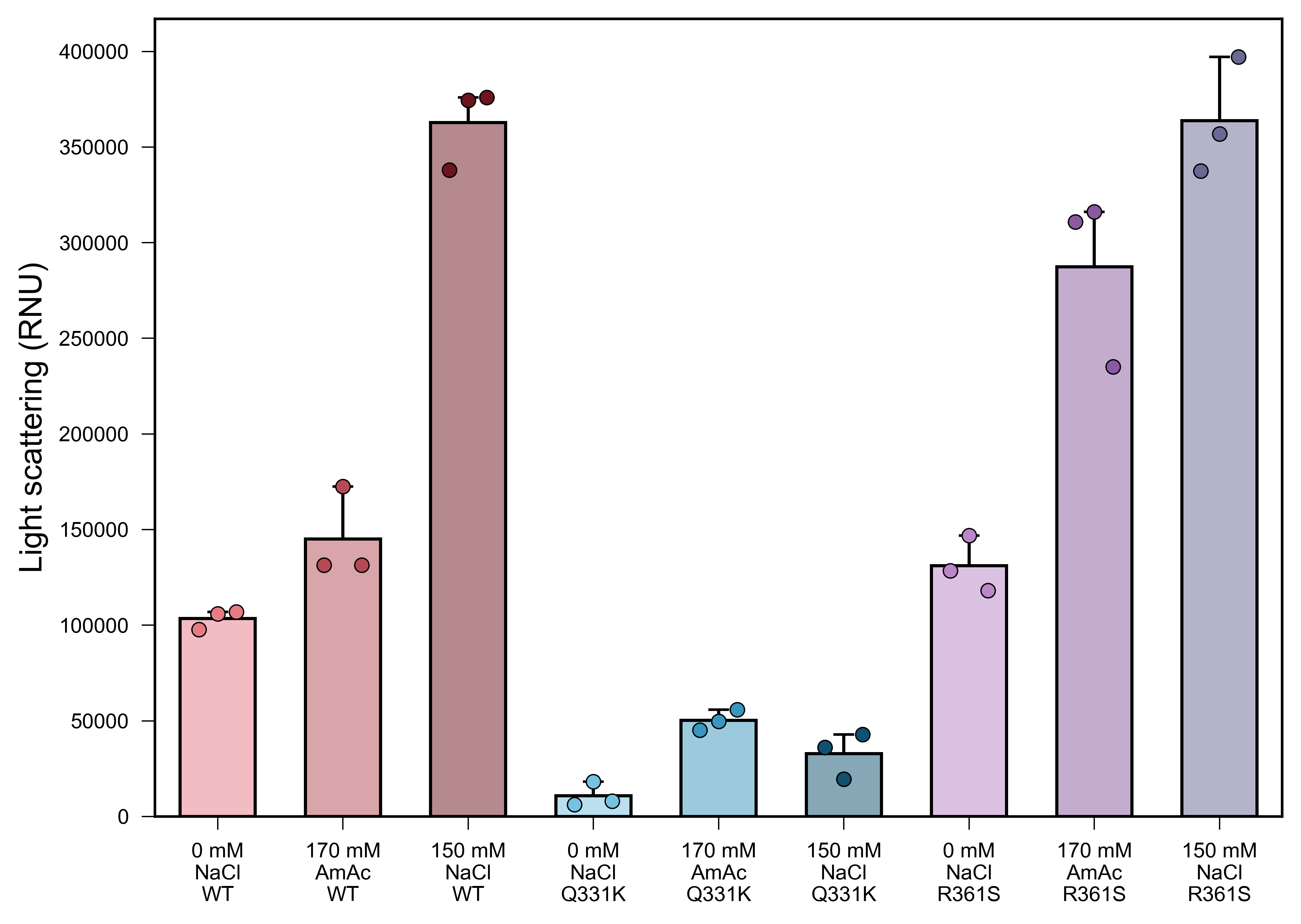
 ***Figure S7. Nephelometry light scattering measurements.*** *Nephelometry values were measured in 20 mM ammonium acetate, 170 mM ammonium acetate or 20 mM ammonium acetate with 150 mM ammonium acetate (n = 3 for the WT, Q331K and R361S TDP-43 CTD).*

*
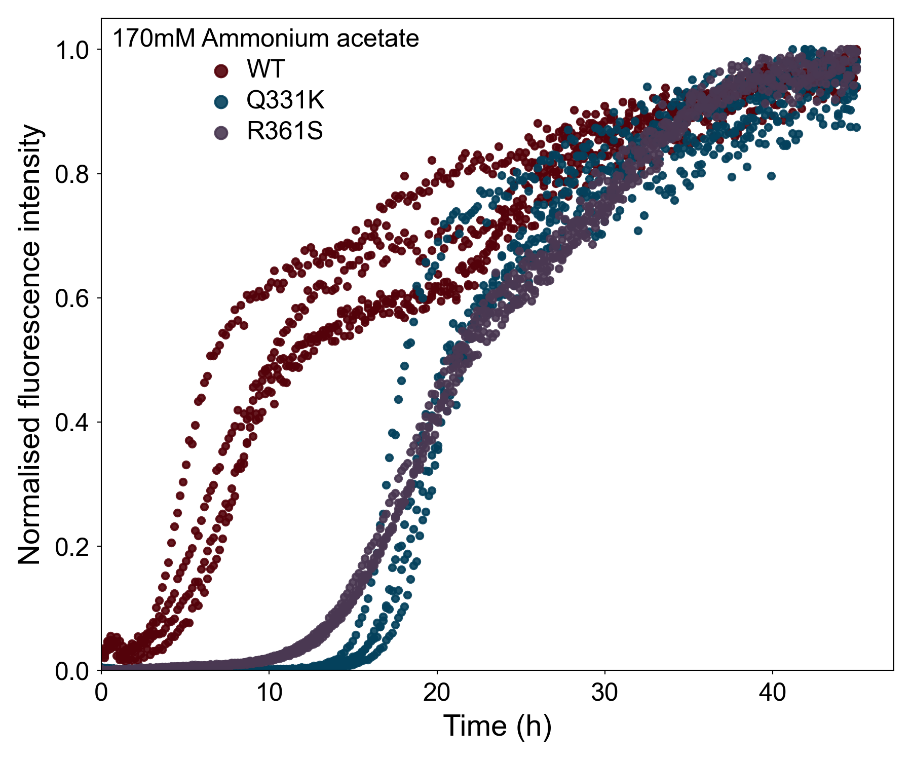
*

***Figure S8. ThT Fluorescence measurements of amyloid formation in 170 mM Ammonium Acetate.*** *ThT fluorescence kinetics of amyloid formation measured in 170 mM ammonium acetate (AmAc), pH 5.5 (n = 4).*

***
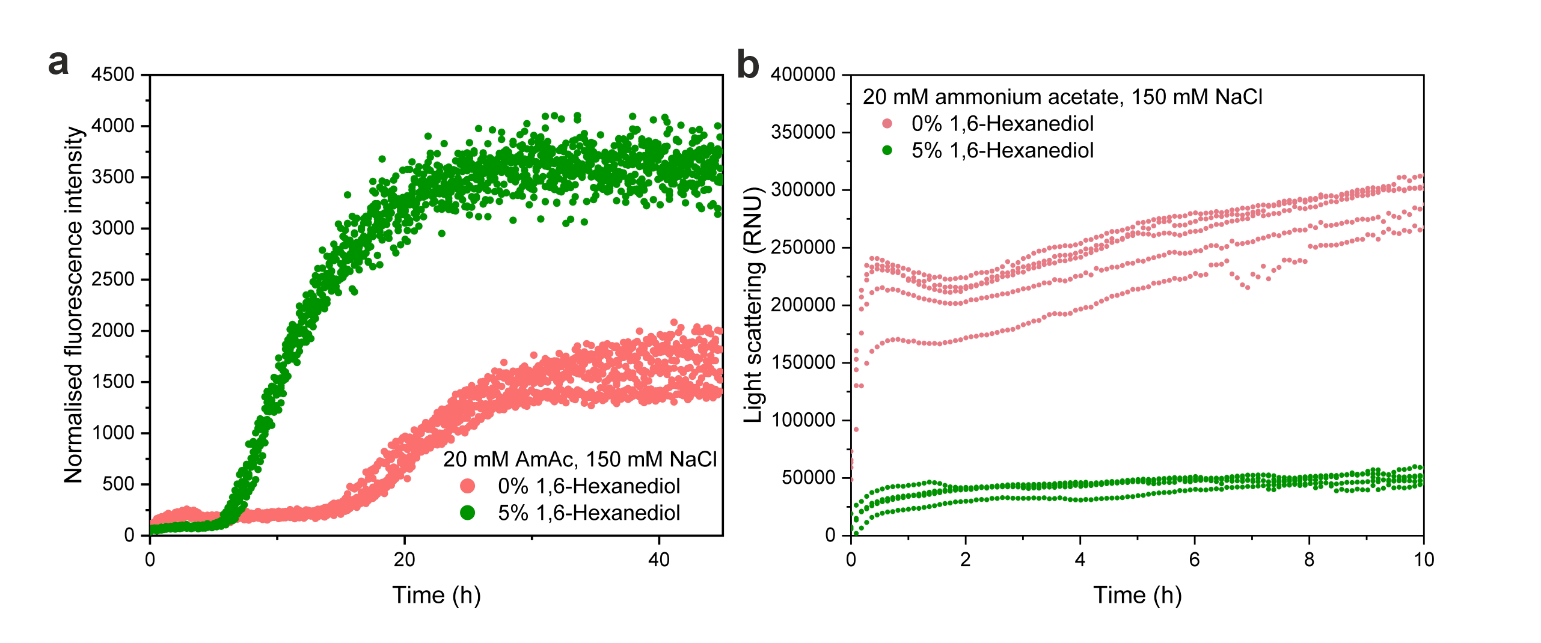
*** ***Figure S9. 1,6-hexanediol disolves biomolecular condensates and accelerates amyloid assembly.*** *(a) ThT fluorescence kinetics of amyloid formation for the WT CTD of TDP-43 (n = 5) in the absence (red) and presence of 5% (v/v) 1,6-hexandediol (green). (b) Nephelometry light scattering measurements (n = 5) for the WT CTD of TDP-43 in the absence (light red) and presence of 5% (v/v) 1,6-hexandediol (green) over a period of 10 hours.*

WT, 150 mM NaCl, 5% 1,6-Hexanediol


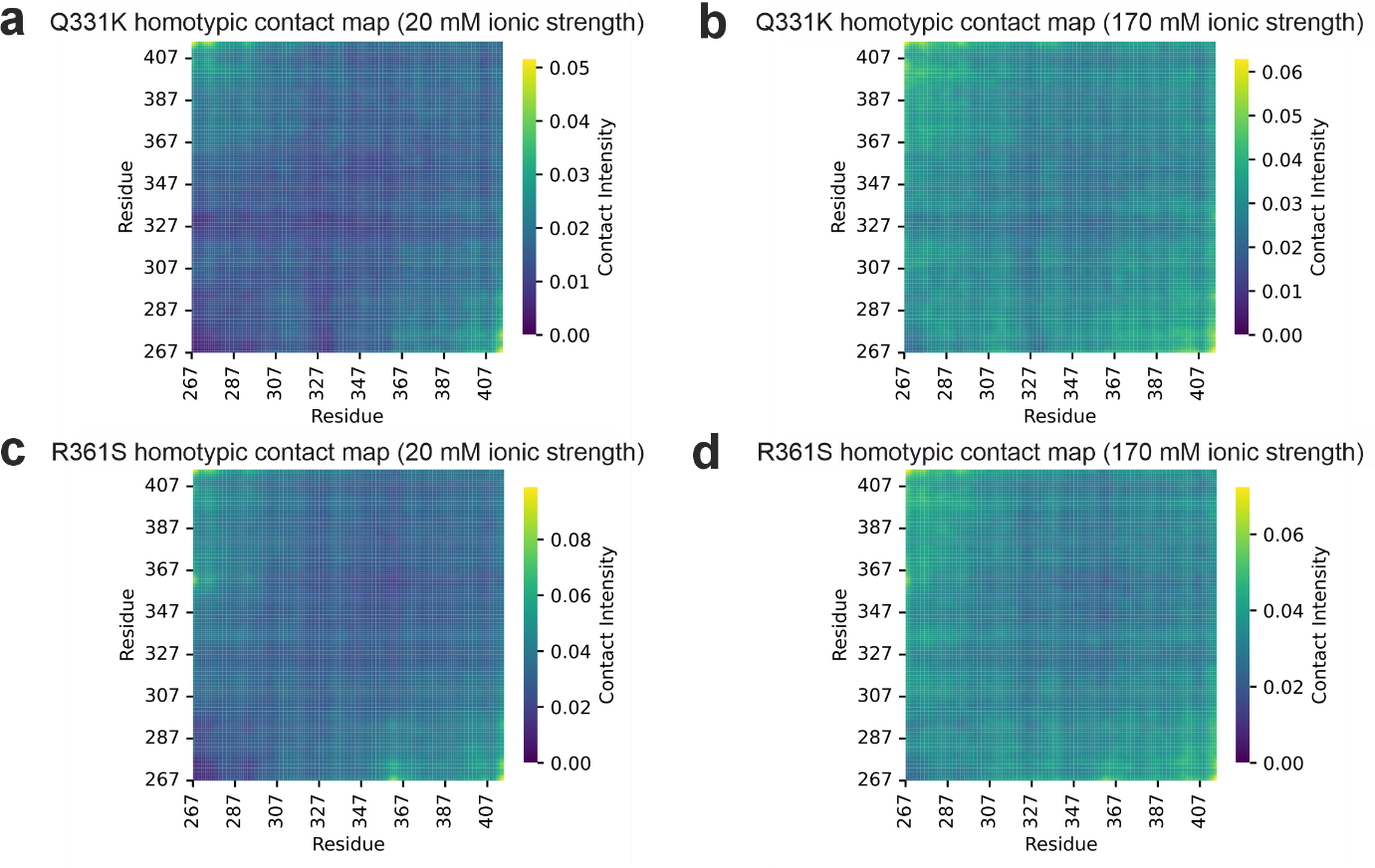


***Figure S10.*** ***CALVADOS simulations map salt-tuned contact networks in TDP-43 CTD variants.*** *Homotypic residue-residue contact maps for Q331K TDP-43 CTD at (a) 20 mM and (b) 170 mM ionic strength and R361S TDP-43 CTD at (c) 20 mM and (d) 170 mM ionic strength.*


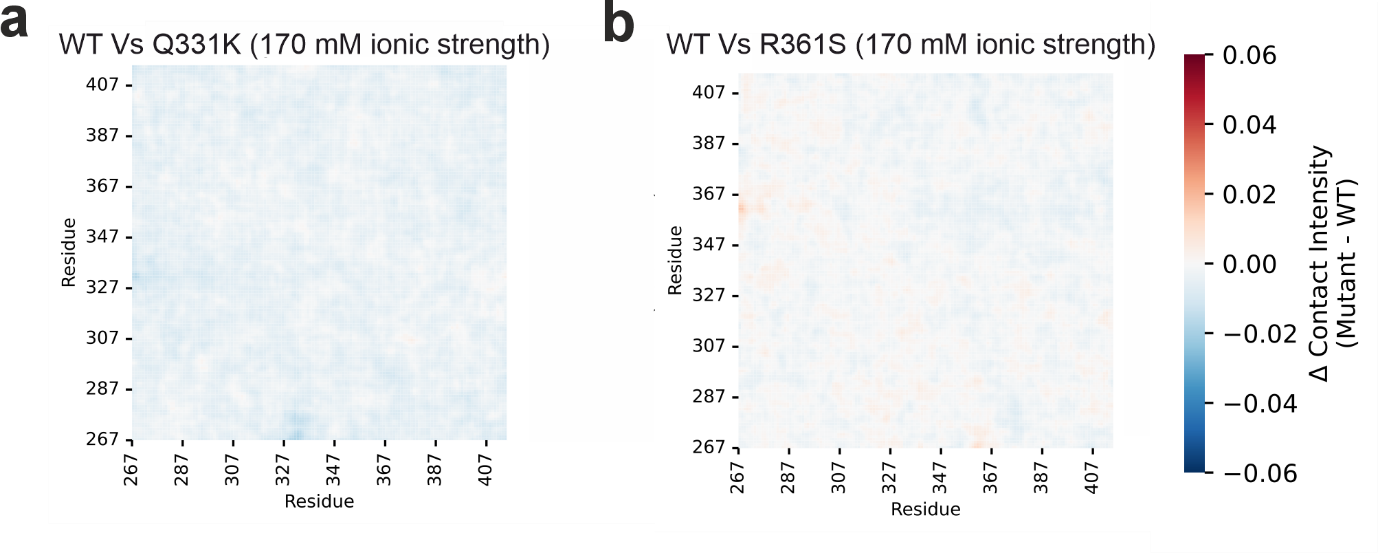


***Figure S11.*** ***Differential homotypic residue-residue contacts maps.*** *Differential homotypic residue-residue contacts maps in 170 mM ionic strength conditions when comparing (a) Q331K with WT TDP-43 CTD and (b) R361S TDP-43 with WT TDP-43 CTD.*

*
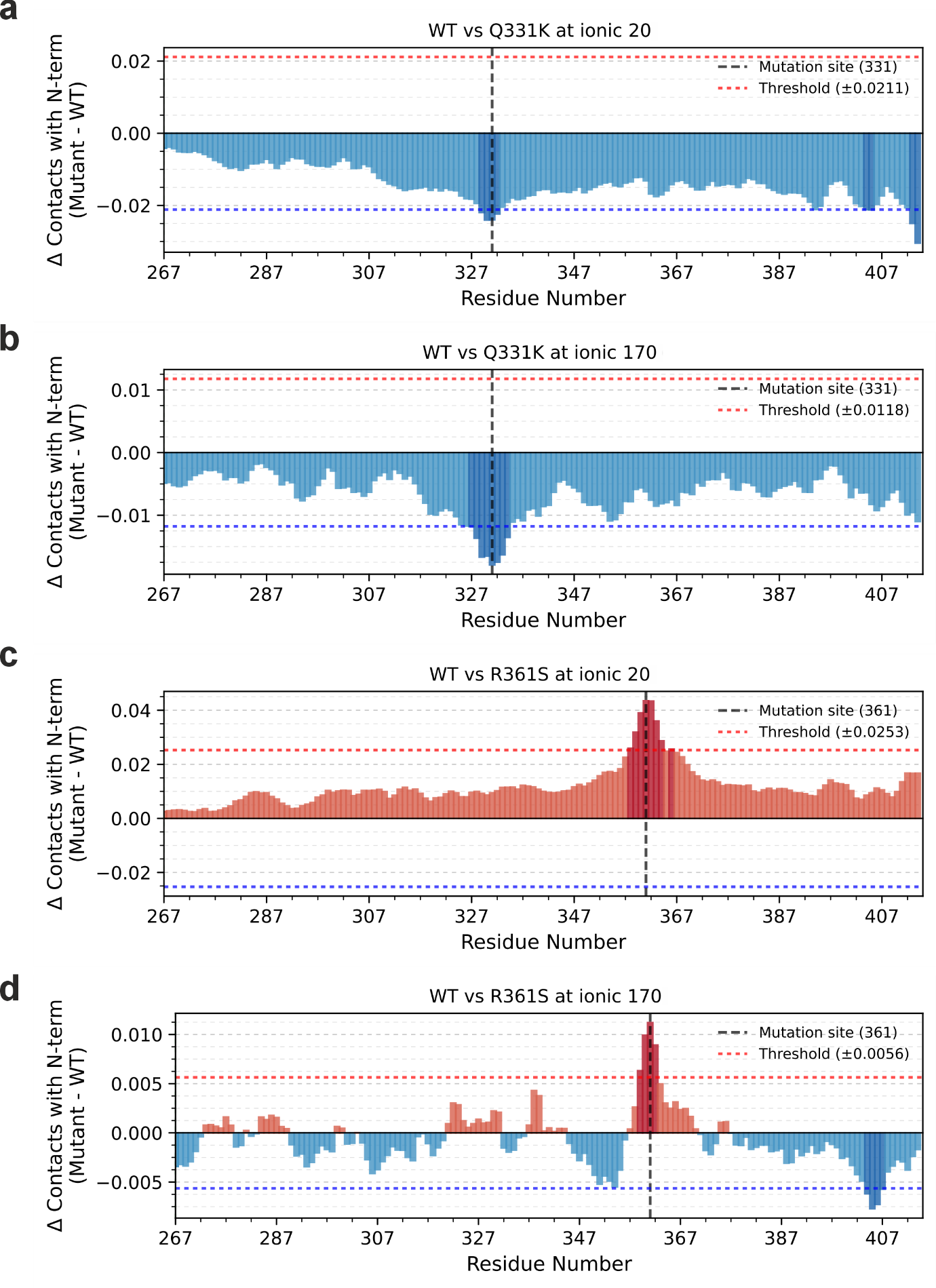
*

***Figure S12. Altered contacts with the N-terminus of TDP-43 CTD in the Q331K and R361S variants under low ionic strength conditions determined using CALVADOS.*** *Calculation of difference in contact intensity between the N-terminus and all other residues of TDP-43 CTD (from Figure 6e,f), when comparing WT TDP-43 CTD with Q331K TDP-43 CTD at 20 mM ionic strength (a) and 170 mM ionic strength (b) and comparing WT TDP-43 CTD with R361S TDP-43 CTD at 20 mM ionic strength (c) and 170 mM ionic strength (d). The residues in the top 5 % of change were identified (blue and red dotted lines). For the Q3331K TDP-43 CTD variant these include residues 327-QAALQSSM-333, M414. For the R361S TDP-43 CTD variant these include residues 359-MQREPNQA-366.*
